# Ykt6-dependent endosomal recycling is required for Wnt secretion in the *Drosophila* wing epithelium

**DOI:** 10.1242/dev.185421

**Published:** 2020-08-14

**Authors:** Karen Linnemannstöns, Leonie Witte, Pradhipa Karuna M, Jeanette Clarissa Kittel, Adi Danieli, Denise Müller, Lena Nitsch, Mona Honemann-Capito, Ferdinand Grawe, Andreas Wodarz, Julia Christina Gross

**Affiliations:** 1 Hematology and Oncology, University Medical Centre Goettingen, Goettingen 37075, Germany; 2 Developmental Biochemistry, University Medical Centre Goettingen, Goettingen 37077, Germany; 3 Molecular Cell Biology, Institute I for Anatomy, University of Cologne Medical School, Cologne 50931, Germany; 4 Cluster of Excellence-Cellular Stress Response in Aging-Associated Diseases (CECAD), Cologne 50931, Germany; 5 Center for Molecular Medicine Cologne (CMMC), University of Cologne, Faculty of Medicine and University Hospital Cologne, 50931 Cologne, Germany

**Keywords:** Wnt secretion, Wnt signalling, Endosomal sorting, Morphogen trafficking

## Abstract

Morphogens are important signalling molecules for tissue development and their secretion requires tight regulation. In the wing imaginal disc of flies, the morphogen Wnt/Wingless is apically presented by the secreting cell and re-internalized before final long-range secretion. Why Wnt molecules undergo these trafficking steps and the nature of the regulatory control within the endosomal compartment remain unclear. Here, we have investigated how Wnts are sorted at the level of endosomes by the versatile v-SNARE Ykt6. Using *in vivo* genetics, proximity-dependent proteomics and *in vitro* biochemical analyses, we show that most Ykt6 is present in the cytosol, but can be recruited to de-acidified compartments and recycle Wnts to the plasma membrane via Rab4-positive recycling endosomes. Thus, we propose a molecular mechanism by which producing cells integrate and leverage endocytosis and recycling via Ykt6 to coordinate extracellular Wnt levels.

## Abstract (precis)

**Summary:** SNARE-dependent secondary secretion via Rab4 recycling endosomes plays an important role in coordinating long-range extracellular levels of the hydrophobic morphogen Wnt/Wingless.

## INTRODUCTION

Cell behaviour and growth is coordinated at the tissue level by morphogen signalling to provide context-specific information in a space-, time- and dose-dependent manner. One such morphogen that forms a concentration gradient across a developing tissue is Wnt. Wnts act on neighbouring and distant target cells to activate Wnt signalling pathways, which play a central role in stem cell maintenance, differentiation in development and adult homeostasis (Nusse and Clevers, 2017). Within the source cells, Wnt trafficking through the secretory pathway is highly regulated to fine-tune extracellular signal distribution. First, Wnts are lipidated in the ER by Porcupine (Kadowaki et al., 1996; Tanaka et al., 2000). This modification is required for their activity and secretion, and is essential for p24 protein-dependent Wnt exit from the ER (Buechling et al., 2011; Port et al., 2011). Here, the cargo receptor Evi [also referred to as Wntless (Wls)] recognizes palmitoleic acid-modified Wnts and escorts them from the ER to the plasma membrane (Herr and Basler, 2012). In the ER, Evi levels depend on Wnt ligands and are regulated by the ERAD pathway (Glaeser et al., 2018). The recycling of Evi from the cell surface to the trans-Golgi network (TGN) enables further transport of newly synthesized Wnts from the TGN to the cell surface. Evi recycling depends both on clathrin-adaptor protein 2 (AP-2)-mediated endocytosis (Gasnereau et al., 2011) and retromer function, because blocking either of these steps leads to a reduction in Wnt secretion (Belenkaya et al., 2008; Harterink et al., 2011; Port et al., 2008; Yang et al., 2008; Zhang et al., 2011). Interestingly, Wnt and Evi only separate in acidified endosomes (Coombs et al., 2010), but the exact routes of post-endocytic trafficking leading to Wnt secretion remain unclear.

Endocytosis into endosomes is required for Wg trafficking, secretion and signalling, as demonstrated in the polarized epithelium of developing *Drosophila* wings (Pfeiffer et al., 2002; Strigini and Cohen, 2000). In addition, there seems to be a dual effect of the endosomal compartment on Wg signalling: impairing early endosomal sorting causes reduction in Wg secretion and signalling (Marois et al., 2006; Seto and Bellen, 2006), whereas blocking endosomal trafficking from late endosome to lysosome increases Wg signalling (Dubois et al., 2001; Seto and Bellen, 2006). Time-course analysis revealed that Wg is first trafficked to the apical membrane and then re-endocytosed before its final secretion. Several hypotheses exist for this postendocytic trafficking: (1) Wg is transcytosed and secreted at the basolateral membrane (Yamazaki et al., 2016); (2) Wg is loaded onto endosome-derived exosomes for export after endocytosis (Gross et al., 2012); and (3) Wg and Frizzled (Fz) receptors meet in endosomal compartments for signalling and degradation (Hemalatha et al., 2016). Therefore, to elucidate the role of Wnt trafficking to endosomal compartments, it is essential to determine whether it is destined for secretion, signalling or degradation.

Previously, we identified the SNARE Ykt6 to be required for the secretion of Wnts on exosomes in *Drosophila* and human cells (Gross et al., 2012). Wnts are secreted on different extracellular vesicles (EVs) such as exosomes (Beckett et al., 2013; Gross et al., 2012; Koles et al., 2012; Menck et al., 2013), e.g. in the context of spermatogenesis and nerve regeneration (Koch et al., 2015; Tassew et al., 2017). Ykt6 is an unusual SNARE, as it lacks a transmembrane domain and therefore cycles between cytosol and membranes (reviewed by Kriegenburg et al., 2019). Ykt6 localizes to different membranes (such as ER, Golgi, endosomal membranes and the plasma membrane) and was found in variable SNARE complexes *in vitro.* In yeast, Ykt6 functions in homotypic fusion of ER and vacuolar membranes, in retrograde Golgi trafficking and in autophagosome formation (Bas et al., 2018; Gao et al., 2018). In higher eukaryotes, Ykt6 seems to play a role in non-canonical autophagosome formation under starvation conditions in human cells (Matsui et al., 2018) and *Drosophila* fat body (Takáts et al., 2018). Considering the ability of Ykt6 to adapt to multiple cellular localizations, we investigate it here as a candidate to orchestrate Wnt secretion from endosomes. Combining *in vivo* genetics, proximity-dependent proteomics and *in vitro* biochemical analyses, we found that cytosol-to-membrane cycling of Ykt6 has an evolutionarily conserved function in endosomal Wnt trafficking in *Drosophila* and in human cells. Ykt6 acts via Rab4 in recycling Wnts to the cell surface, and we propose that this is a novel mechanism for fine-tuning of Wnt secretion in endosomes.

## RESULTS

### Loss of Ykt6 blocks Wnt secretion

**Fig. 1.**
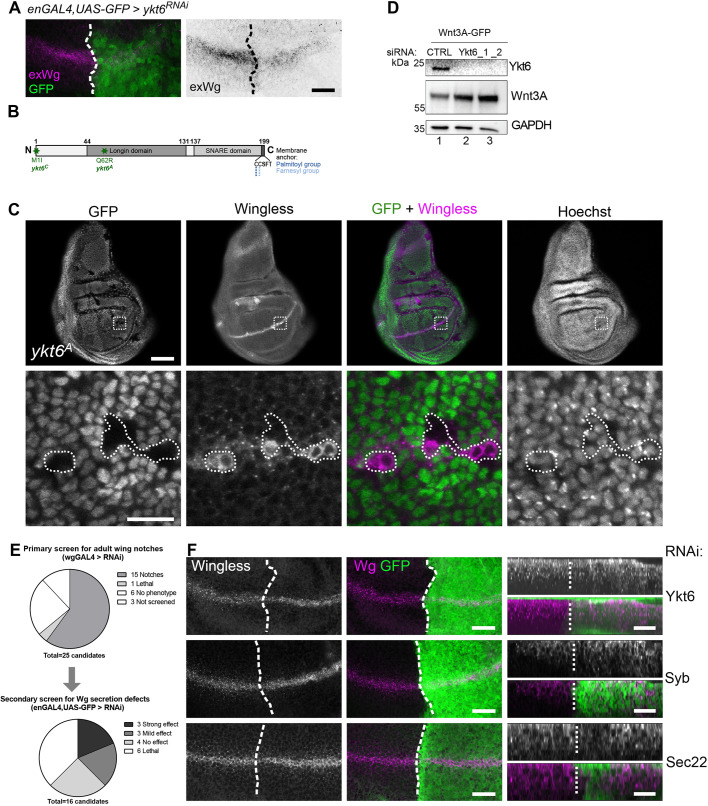
**Loss of Ykt6 blocks Wnt secretion.** (A) RNAi-knockdown of Ykt6 in the posterior compartment of third instar WIDs marked by co-expression of GFP (engrailed-Gal4, UAS-GFP/UAS-ykt6RNAi) causes extracellular Wingless reduction. The GFP-negative anterior compartment serves as an internal control. Maximum intensity projection of 20 sections (distance 0.5 µm) depicted for visualization. (B) Scheme of *Drosophila ykt6* mutant alleles. (C) Wingless protein accumulates in *ykt6^A^* clones marked by the absence of GFP. The lower panels depict enlarged images of the areas outlined in the upper panels. (D) Western blot analysis of intracellular Wnt accumulation in Hek293T cells transfected with control or Ykt6-1 or Ykt6-2 siRNA. (E) RNAi against 25 *Drosophila* SNAREs was screened for Wnt secretion defects in adult wings (wgGAL4) and third instar WIDs (enGAL4) (see also Table S1). (F) Knockdown of Ykt6 and Syb by RNAi in the posterior compartment of third instar WIDs marked by co-expression of GFP leads to intracellular Wg accumulation, whereas Sec22 does not affect Wg distribution. The GFP-negative compartment serves as an internal control. Left panels in F show maximum intensity projections of six (Ykt6), 15 (Syb) and two (Sec22) sections (distance 1 µm) depicted for visualization. Right panels in F show optical transverse sections. Scale bars: 20 µm.

To analyse the role of Ykt6 in Wnt secretion, we used the polarized epithelium of *Drosophila* wing imaginal discs (WIDs), a well-established model system to study the secretory pathway of Wingless (Wg), the *Drosophila* homologue of Wnt1 (reviewed by Parchure et al., 2018; Swarup and Verheyen, 2012). RNAi-mediated knockdown of Ykt6 in third-instar WIDs strongly reduced extracellular Wg staining (Fig. 1A; Gross et al., 2012), indicating a block of Wg secretion. We confirmed this RNAi phenotype using two available loss-of-function alleles: *ykt6^C^*, which has a mutated start codon (M1I); and *ykt6^A^*, which carries a Q62R exchange in the Longin domain (Haelterman et al., 2014) (Fig. 1B). These alleles are homozygous lethal, confirming the essential role of Ykt6 described in yeast (McNew et al., 1997). GFP-negative *ykt6^A^* mutant mitotic clones were small compared with control clones (Fig. S1A), yet Wg accumulated intracellularly within these clones, as observed for RNAi (Fig. 1C). DE-Cadherin staining was unaffected in *ykt6^A^* mutant clones, indicating that cargo trafficking from the ER through the Golgi to the plasma membrane is unperturbed (Fig. S1B). This implies that Ykt6 is required for Wg secretion at a post-Golgi step.

To confirm these findings*,* we next investigated the role of Ykt6 in human cells. Ykt6 knockdown in human Hek293T cells caused intracellular accumulation of overexpressed Wnt3A-GFP (Fig. 1D) and reduced endogenous Wnt5A secretion from SK-BR-3 breast cancer cells (Fig. S1C). Thus, the role of Ykt6 in Wnt secretion appears to be evolutionarily conserved. Proteins of the SNARE family drive membrane fusion by formation of a trans-SNARE complex consisting of four specific v- and t-SNAREs present at vesicle and target membranes. Different trafficking steps are mediated by preferential sets of SNAREs to ensure a directional flow of membranes and cargo (Dingjan et al., 2018). However, Ykt6 has multiple sites of action and it has been shown to interact with different SNARE partners *in vitro* (Tsui et al., 2001). To understand at which step Ykt6 is involved in post-Golgi Wnt trafficking, we undertook a comparative RNAi candidate approach in *Drosophila* WIDs, comparing its knockdown with the knockdown of early and late secretory SNAREs (Fig. 1E, Table S1). First, the adult wings of wgGal4-driven RNAi crosses of all 25 SNAREs were analysed for Wnt signalling defects, i.e. wing notches (Fig. 1E, upper diagram). Owing to the general importance of membrane fusion events for protein secretion (Gordon et al., 2010), 15 of those 25 SNAREs showed notches and one cross was lethal (Table S1). Next, enGal4-driven RNAi of those 16 was analysed in WIDs for Wg secretion defects by comparing and visually scoring Wg staining in the anterior and the posterior compartment (Fig. 1E, lower diagram). Under those conditions, six candidates were lethal and six affected Wg secretion. Golgi SNAREs, such as Syx5 and Bet1, strongly reduced Wg secretion and overall cell survival, and were not further investigated. Sec22 and Vamp7 contain a Longin domain like Ykt6 and, together with Synaptobrevin (Syb), act in plasma membrane fusion of secretory vesicles (Gordon et al., 2017) and Wg secretion (Gao et al., 2017; Li et al., 2015; Yamazaki et al., 2016). Indeed, we observed Wg accumulation and wing notches for Sec22 and Syb, but not for Vamp7 (Table S1). Transverse optical sections clearly showed that Syb RNAi leads to apical accumulation of Wg, similar to the phenotype observed with Ykt6 (Fig. 1F, middle panel). As Ykt6 negatively interacts with Syb and Sec22 in *Drosophila* cells (Gordon et al., 2017), we asked whether Ykt6 knockdown would affect these late secretory SNAREs *in vivo*. Staining for Sec22, Syb and Vamp7 in enGAL4/Ykt6-RNAi WIDs revealed that Ykt6 depletion affects neither localization nor stability of these three SNAREs *in vivo* (Fig. S1D). We further tested for a role for these SNAREs in Wnt secretion and signalling in non-polarized Hek293T cells. In an autocrine Wnt reporter assay, knockdown of Ykt6 and VAMP1 (human Syb homologue) reduced Wnt activity, whereas Sec22B and VAMP7 did not (Fig. S1E). Taken together, these data suggest that Ykt6-mediated trafficking events resemble those of Syb, a SNARE previously described in a post-endocytic step in Wg secretion in WIDs (Yamazaki et al., 2016).

### Ykt6 acts on endosomal compartments after apical presentation

**Fig. 2.**
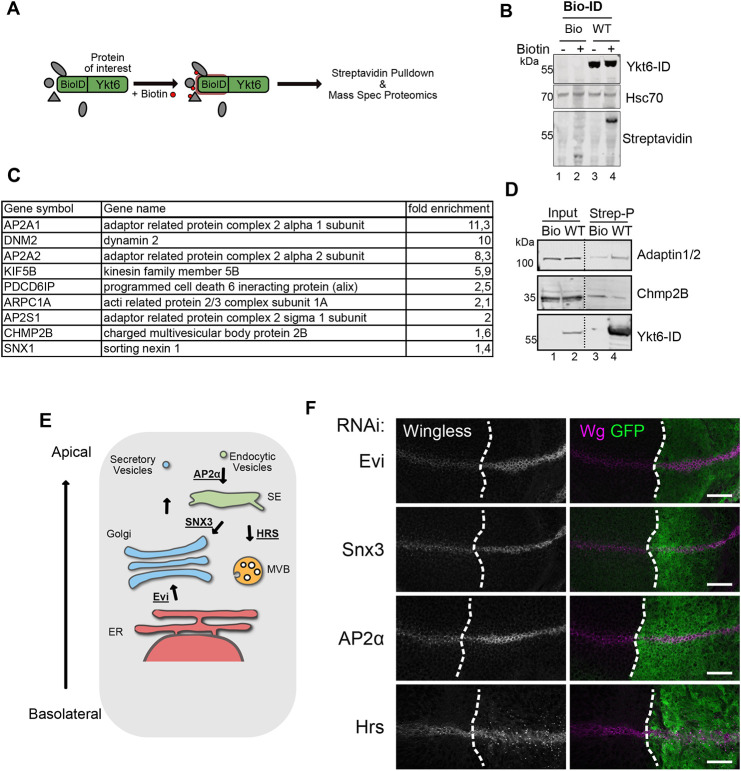
**Ykt6 acts on endosomal compartments after apical presentation.** (A) Scheme of BioID labelling in Hek293T cells: Ykt6 was N-terminally tagged with a BioID domain. Upon addition of biotin, a streptavidin pull-down was performed, and control and Ykt6-WT samples were subjected to proteomics identification. (B) Western blot of biotin labelling of Ykt6-BioID and control in the presence of 50 µM biotin. All proteins identified by mass spectrometry in two independent experiments (significance level *P*=0.003 and 1.3-fold over BioID control samples) are listed in Table S2. (C) Enrichment scores for BioID-identified proteins of the endocytic pathway. (D) Western blot of Ykt6-WT-mediated BioID labelling of AP2A1/2 and Chmp2B. (E) Overview of Wnt secretion components involved after initial apical plasma membrane presentation of Wg. (F) Wg accumulation phenotypes of different factors required for Wg secretion. RNAi against Evi, Snx3 and AP2α expressed with enGAL4, and RNAi against Hrs and Dcr expressed with UAS-Dcr; enGAL4. Images represent a single confocal section and are representative of more than six WIDs per RNAi from three independent experiments. Scale bars: 20 µm.

To clarify the direction of Ykt6-mediated trafficking events, we used an unbiased BioID approach to label proteins in close proximity (Roux et al., 2012, 2018) and thereby identify potential Ykt6 interaction partners informative of Ykt6 sub-endosomal localization. Ykt6 was N-terminally tagged with the prokaryotic BirA* domain. This promiscuous ligase biotinylates amine groups of neighbouring proteins within a 10 nm radius upon addition of biotin. Wild-type (WT) and mock constructs were expressed in human Hek293T cells in the presence of 50 µM biotin; biotinylated proteins were purified by streptavidin pulldown and subjected to mass spectrometry (Fig. 2A,B). We identified a total of 143 biotinylated proteins enriched over background in cells expressing Ykt6-WT (Table S2). In general, BioID captures weak and transient protein-protein interactions and proximate proteins (Liu et al., 2018). Reactome functional network (Gobert et al., 1996) and Kegg pathway analysis (Kanehisa et al., 2016) of identified proteins connected Ykt6 to processes like vesicle trafficking, metabolic processes and endocytosis (Fig. S2A-C). These connections are in line with the pleiotropic effects observed for Ykt6 in diverse membrane-associated processes such as ER-Golgi traffic (Fukasawa et al., 2004; McNew et al., 1997; Zhang and Hong, 2001), autophagy (Bas et al., 2018; Gao et al., 2018; Matsui et al., 2018; Takáts et al., 2018) and plasma membrane fusion (Gordon et al., 2017). However, we did not identify other SNAREs using the BioID approach, potentially owing to the long labelling time of first generation BioID constructs (Roux et al., 2018).

Interestingly, and supporting the findings from the WID candidate screen, we found nine candidates connected to endocytosis (Fig. 2C). Among them are both early (Clathrin adaptor AP2 complex components and Dynamin2) and late (Alix and Chmp2B) endosomal proteins. We confirmed Ykt6-mediated BioID labelling of AP2A1/2 by immunoblotting of streptavidin pulldown from Hek293T cell lysates (Fig. 2D). Furthermore, knockdown of Dynamin 2, Chmp2B and Alix in Hek293T Wnt reporter cells reduced autocrine Wnt signalling activity (Fig. S2D). Together with the results from the SNARE *in vivo* RNAi approach, this supports a connection between Ykt6 and endosomal sorting in Wnt signalling.

Last, the identification of AP2 in the BioID approach and the similarity to the Syb phenotype prompted us to compare Ykt6 knockdown with depletion of different Wnt secretion components involved after apical plasma membrane presentation of Wg (Fig. 2E,F). Similar to Ykt6 and Syb RNAi, depletion of Evi, SNX3 and AP2α complex components led to Wg accumulation close to the membrane. In contrast, knockdown of the multivesicular body (MVB) component Hrs displayed punctate accumulation in Wg-secreting and -receiving cells (Fig. 2E,F). We therefore hypothesized that Ykt6 might be either involved in recycling of the transmembrane protein Evi or secretion of Wg from endosomal compartments.

### Ykt6 knockdown is not sufficient to block Evi recycling

**Fig. 3.**
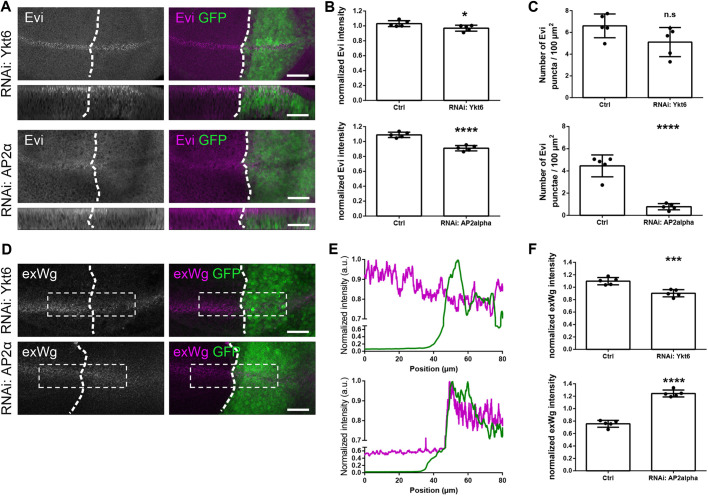
**Ykt6 knockdown is not sufficient to block Evi recycling.** (A-C) RNAi against Ykt6 and AP2α was expressed with enGAL4,UAS-GFP, and stained for Evi. (A) The upper panels depict a maximum intensity projection of 15 apical *xy* sections (distance 1 µm); the lower panels are a transverse *xz* section of 20 pixels. (B) Comparison of Evi fluorescence intensity in *n*=5 biologically independent samples. Data are mean±s.d., *P=0.0425, ****P<0.0001. (C) Quantification of Evi puncta in *n*=5 biologically independent samples. Data are mean±s.d., ****P<0.0001. (D-F) RNAi against Ykt6 and AP2α was expressed with enGAL4 and stained for extracellular Wg. (D) A maximum intensity projection of all sections covering the entire apico-basal axis is depicted for visualization. (E) Profile of the extracellular Wg staining in the ROI depicted in D of the corresponding average intensity projection. This compares exWg in the anterior (control, no GFP) with the posterior (RNAi, GFP-positive) region for this one representative example. (F) Comparison of exWg fluorescence intensity in *n*=5 biologically independent samples. Data are mean±s.d., ***P=0.0007, ****P<0.0001. Scale bars: 20 µm.

Similar to Evi knockdown, SNX3 knockdown leads to Wg accumulation. In the absence of all Retromer components (VPS26, VPS35 and SNX3) Evi is lysosomally degraded, instead of retrogradely transported towards the Golgi (Belenkaya et al., 2008; Franch-Marro et al., 2008; Port et al., 2008; Yang et al., 2008). AP2 is crucial for the endocytosis of membrane proteins such as Evi, as AP2α RNAi strongly reduces apical Evi staining (Belenkaya et al., 2008; Franch-Marro et al., 2008; Port et al., 2008; Yang et al., 2008). In contrast, we found that Ykt6 knockdown had only a weak effect on Evi (Fig. 3A-C), thus making a function of Ykt6 in Evi recycling unlikely. This is in line with a model from human cell culture, in which Wnt and Evi separate after reaching acidified endosomes (Coombs et al., 2010). If this is correct, then we expect AP2α and Ykt6 knockdown to differentially affect extracellular Wg. Indeed, staining of non-permeabilized WIDs revealed Wg accumulation at the apical surface in AP2α RNAi, whereas extracellular Wg levels were reduced upon loss of Ykt6 (Fig. 3D-F). Moreover, Wg endocytosis was unchanged in a pulse-chase Wg antibody uptake assay in Ykt6 knockdown compared with control (Fig. S3). Taken together, this demonstrates that Clathrin-mediated endocytosis and Retromer sustain the Evi recycling route. In contrast, Ykt6-dependent trafficking appears to be necessary for postendocytic secondary secretion of Wg independent of Evi.

### Ykt6 acts on Wnt trafficking at the level of endosomes

**Fig. 4.**
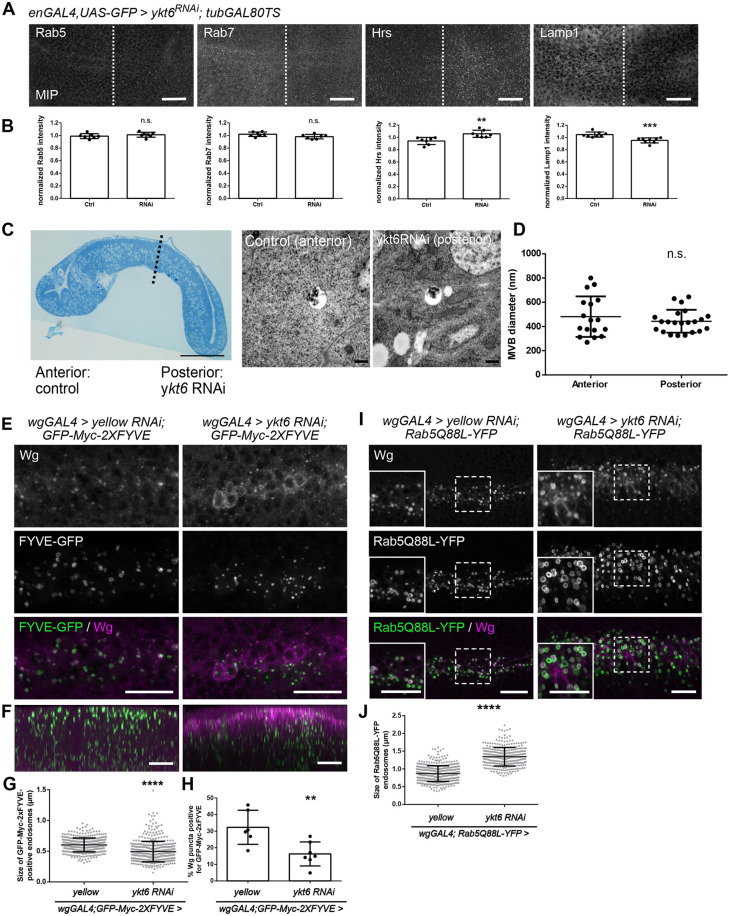
**Ykt6 acts on Wnt trafficking at the level of endosomes.** (A) Localization of different organelle markers in wild type (left) and Ykt6 RNAi (right) from enGAL4, UAS-GFP/Ykt6 RNAi WIDs. Maximum intensity projections of 13 (Rab5), seven (Rab7, Lamp1) and nine (Hrs) sections (distance 1 µm) depicted for visualization. Scale bars: 20 µm. (B) Quantification of fluorescence intensity in *n*=7 (Rab5, Rab7 and Hrs) or *n*=8 (Lamp1) biologically independent samples from A. Data are mean±s.d., **P=0.0026, ***P=0.0004. (C) Semi-thin section of a WID (left) and electron microscopy images of MVBs in a WID (right) during time-controlled depletion of Ykt6 by RNAi (engrailed-Gal4, UAS-GFP/UAS-ykt6RNAi; tubGal80-TS/+ larvae reared for 3 days at 29°C). Scale bars: 50 μm (left); 500 nm (right). (D) Quantification of MVB size in electron microscopy images from cells in the anterior (*n*=17) and posterior (*n*=22) compartments of WIDs. (E,F) GFP-Myc-FYVE was expressed with wgGAL4 and yellow (control) or ykt6 RNAi to label PI(3)P-containing endosomes. (E) MIP of six apical sections (distance 0.5 µm). (F) Transverse *xy* section. Scale bars: 10 µm. (G) Quantification of E. The diameter of GFP-Myc-FYVE -positive vesicles with a clear lumen was measured. Four representative WIDs from three biological replicates with in total 394 (yellow RNAi) and 460 (ykt6 RNAi) enlarged endosomes were quantified. Data are mean±s.d., ****P<0.0001. (H) Quantification of F. The number of Wg puncta positive for GFP-Myc-2XFYVE was quantified. Data are mean±s.d., **P=0.0071. (I) Constitutively active Rab5Q88L-YFP was expressed with wgGAL4 and yellow (control) or ykt6 RNAi. Images represent a single confocal section. Scale bars: 10 µm. (J) Quantification of I. The diameter of Rab5Q88L-YFP-positive vesicles with a clear lumen was measured. Five representative WIDs from three biological replicates with, in total, 446 (yellow RNAi) and 326 (ykt6 RNAi) enlarged endosomes were quantified. Data are mean±s.d., ****P<0.0001.

As Ykt6 appears to function after Evi and Wg separate from each other, we hypothesized that it mediates an endosomal fusion event. Upon Ykt6 RNAi, we observed no change in staining for early (Rab5) or late (Rab7) endosomal markers, but a slight increase of Hrs and a slight decrease in staining for Lamp-1, a marker for lysosomes (Fig. 4A,B). Hrs captures ubiquitylated proteins and recruits ESCRT-I to sort cargo into MVBs for degradation or cargo sorting onto exosomes, but recently Hrs was also implicated in promoting the recycling of cargo via WASH-actin (MacDonald et al., 2018). Interestingly, *ykt6^A^* and *ykt6^C^* homozygous lethality can be rescued by removing one copy of *hrs^D28^*, indicating that *ykt6* and *hrs* genetically interact (Fig. S4A). Next, we analysed MVB morphology and the formation of intraluminal vesicles, which can be secreted as exosomes, a population of small extracellular vesicles, in an ESCRT-dependent and Alix-Syntenin-regulated manner (Baietti et al., 2012). In electron microscopy sections of WIDs, MVBs were of similar sizes in wild-type and Ykt6 RNAi compartments (Fig. 4C,D), and the apical membrane showed no strong morphological defects upon Ykt6 loss (Fig. S4B). This indicates that Ykt6 knockdown does not impair MVB morphology.

Hrs is recruited to endosomes via its Fab1/YOTB/Vac1/EEA1 (FYVE) domain, which interacts with locally generated phosphatidylinositol 3-phosphate (PI3P) (Urbé et al., 2002). To check whether increased binding of Hrs to endosomes in Ykt6 KD cells was due to a change in the composition of PI3P, which is abundant in early endosomes and MVBs, we used 2xFYVE-GFP to mark PI3P-containing endosomes *in vivo* (Wucherpfennig et al., 2003). In larval wing disc cells, 2xFYVE-GFP mostly localizes to Rab7- but not to Rab5- or Rab11-positive endosomes (Abe et al., 2009). In control WIDs, Wg-expressing cells showed a ∼32% colocalization of Wg with FYVE-GFP in puncta, compared with only 16% of Wg in Ykt6 knockdown (Fig. 4E-H). FYVE-GFP structures were smaller in Ykt6 RNAi compared with control and Wg accumulated intracellularly at the plasma membrane (Fig. 4E-H and Fig. S4C), indicating that lack of Ykt6 reduces the pool of late PI3P-containing endosomes.

To check whether Ykt6 mediates an endosome-to-plasma membrane fusion event, we used a constitutively active Rab5 (Rab5Q88L) to enlarge and visualize endosomes (Zhang et al., 2007). WgGal4-driven Rab5Q88L-YFP expression in WIDs led to enlarged endosomes positive for endogenous Wg (Fig. 4I, left panel). In Ykt6-RNAi WIDs, these endosomes were significantly larger and, in addition, Wg was seen outside Rab5Q88L endosomes close to the membrane (Fig. 4I, right panel, J), similar to Ykt6 KD alone (Fig. S4D). Higher resolution using Airyscan imaging revealed that Wg accumulates mainly laterally below junctions as marked with DE-Cadherin (Fig. S4E). Rab5Q88L-YFP expression alone did not impair Wg secretion and signalling, as wings developed normally, but when this was combined with Ykt6-RNAi it resulted in pupal lethality (Fig. S4F). It is noteworthy that we did not find endosomal size alterations in cells that overexpressed a WT version of Rab5-YFP (Fig. S4G,H). Colocalization of Wg- and endogenous Rab5 was significantly decreased in Ykt6 knockdown (Fig. S4I), further confirming Rab5Q88L results. Taken together, our *in vivo* genetic analyses demonstrate that lack of Ykt6 decreases Wg trafficking to late endosomes, possibly because Ykt6-dependent, endosome-derived vesicles accumulate close to the plasma membrane. Thus, Ykt6 is involved in endosomal Wg trafficking required for Wnt release.

### The Ykt6 SNARE domain is required for cycling between compartments

**Fig. 5.**
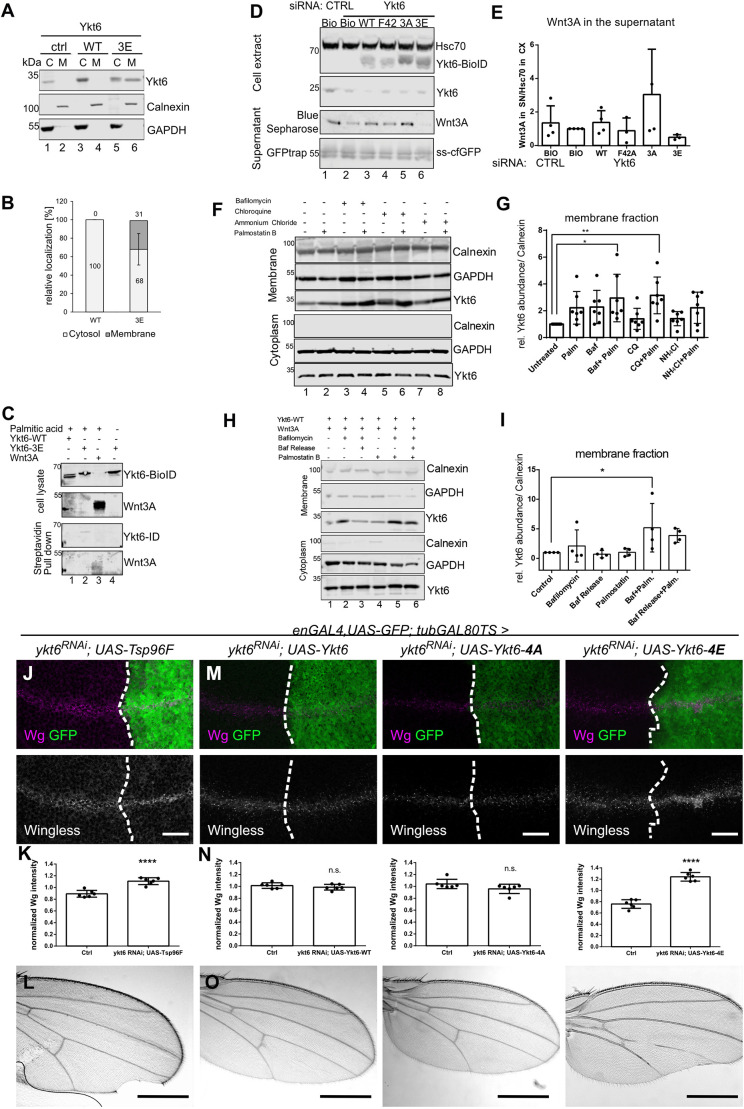
**A Ykt6 SNARE domain is required for cycling between compartments.** (A,B) Representative blot (A) and quantification (B) of detergent fractionation of Hek293T cells transfected with Ykt6-WT and Ykt6-3E constructs. C, cytoplasmic; M, membrane fraction, *n*=3. (C) Click palmitoylation assay of Ykt6-WT and Ykt6-3E; Wnt3A is a positive control. Representative blot of three biological replicates. (D,E) Representative blot (D) and quantification (E) of Wnt3A secretion from Hek293T cells transfected with Ykt6-WT, phosphor-mutant Ykt6-3A and Ykt6-3E, and Longin domain mutant Ykt6-F42 constructs. *n*=4. One-way ANOVA (no significant differences). (F,G) Inhibiting endosomal acidification and depalmitoylation affects Ykt6 subcellular localization. (F) Representative blot of cell fractionation of untagged Ykt6 mutant constructs in Hek293T cells, treated with bafilomycin, chloroquine or ammonium chloride in combination with palmostatin B, stained for Ykt6 and fraction markers. (G) Quantification of Ykt6 in the membrane fraction, *n*=7; *P=0,01, **P=0.005 one-way ANOVA. (H,I) Ykt6 membrane recruitment and release. Blot of cell fractionation of untagged Ykt6 in Hek293T cells treated with bafilomycin and palmostatin B, and release from bafilomycin inhibition stained for Ykt6 and fraction markers. (I) Quantification of H from *n*=4; *P=0,03, one-way ANOVA. (J-L) Time-controlled depletion of Ykt6 by RNAi (engrailed-Gal4, UAS-GFP/UAS-ykt6RNAi; tubGal80-TS/UAS-Tsp96F larvae reared for 3 days at 29°C) causes intracellular Wg accumulation (J,K) and wing notches (L). (M-O) Time-controlled Ykt6 RNAi-induced block of Wg secretion and adult wing margin notches can be rescued by co-overexpression of wild-type Ykt6 and the SNARE mutant Ykt6-4A (left and middle panels), but not by Ykt6-4E (right panels). (K,N) Quantification of fluorescence intensity in *n*=6 biologically independent samples from J,M. Data are mean±s.d., ****P<0.0001. (J,M) Projections of six subapical sections (distance 1 µm, J) and six subapical sections (distance 0.5 µm, M). Representative images of more than 10 discs from *n*=3. Scale bars: 20 µm (J,M); 500 µm in adult wing images (L,O).

In contrast to SNAREs with a transmembrane domain, Ykt6 is able to cycle from cytosol to membranes and back owing to its reversible C-terminal palmitoylation. Depalmitoylation of Ykt6 was described to prevent its sorting into MVBs and consequently its inactivation (Meiringer et al., 2008). To understand how Ykt6 membrane recruitment mediates Wnt secretion, we mutated the SNARE domain of Ykt6 to prevent interactions with other SNARE partners and therefore fusion events. As shown for VAMP8, mutation of serine/threonine residues to glutamic acid in the SNARE layers facing each other inhibits fusion of secretory granules by sterically blocking the interaction of the SNARE helices (Malmersjö et al., 2016). Within the SNARE layers of human Ykt6, we mutated three serine residues to glutamic acid (Ykt6-3E) and structural modelling showed steric hindrance of these glutamic acids with the auto-inhibited state of Ykt6 (Fig. S5A,B). In this state, the Longin domain binds to the farnesyl group at the C terminus, bringing it into close proximity to the SNARE domain (Tochio, 2001). We therefore investigated the intracellular localization of these Ykt6 constructs in Hek293T cells. Overexpressed Ykt6-WT showed strong cytoplasmic staining partially overlapping with Calnexin, whereas the mutated SNARE Ykt6 (Ykt6-3E) was more punctate at Golgi and the plasma membrane, suggesting accumulation at membranes (Fig. S5C-F). To confirm this biochemically, we separated cytosolic and membrane-bound proteins by differential detergent fractionation (Baghirova et al., 2015). Indeed, overexpressed Ykt6-3E was found in the membrane fraction, whereas overexpressed Ykt6-WT and endogenous Ykt6 was mostly detected in the cytoplasmic fraction (Fig. 5A,B). We hypothesized that Ykt6-3E attached more stably to membranes because it was unable to fold and release the palmitoylation. To address this possibility, we monitored the steady-state level of palmitoylated Ykt6-WT and -3E in a click-palmitate assay (Haberkant et al., 2016). In the pull down of all palmitoylated proteins, Wnt3A, as a positive control, and Ykt6-3E were both detected, whereas Ykt6-WT was below the detection limit (Fig. 5C). This indicates that the majority of Ykt6-WT reverts into its autoinhibited, depalmitoylated form in the cytoplasm, whereas depalmitoylation of Ykt6-3E is hindered and therefore some remains associated with membranes. This is in line with findings in yeast, where the release of Ykt6 from membranes into the cytoplasm depends on a functional Longin domain and its intramolecular interaction with the SNARE domain to fold into a soluble, closed conformation (Fukasawa et al., 2004; Tochio, 2001). Expressing siRNA-resistant, N-terminally-tagged Ykt6 mutant constructs in Hek293T cells, we found that, in contrast to Ykt6-WT and a non-phosphorylatable Ykt6-3A, Ykt6-3E is unable to rescue Wnt secretion (Fig. 5D,E). Mutation of F42 to alanine, a site within the Longin domain and required for the cytoplasmic, closed conformation of Ykt6 (Tochio, 2001), did not reduce Wnt secretion. All these constructs did not affect secretion of secreted GFP (ssGFP; Suzuki et al., 2012), indicating that the SNARE domain is functionally involved in Wnt secretion (Fig. 5D,E).

Endosomes acidify during trafficking towards the perinuclear region (Wallroth and Haucke, 2018), but also during trafficking towards the plasma membrane, as passage through an acidic compartment is required for Wnt secretion (Coombs et al., 2010). To understand how Ykt6 changes from the auto-inhibited soluble form into the membrane-bound active form, we tested how blocking depalmitoylation by Palmostatin B and acidification by Bafilomycin A1, chloroquine or ammonium chloride affect Ykt6 recruitment to membranes. To increase the detection limit of endogenous Ykt6, proteins in membrane and cytoplasmic fractions were methanol precipitated (Wessel and Flügge, 1984). Bafilomycin A1, chloroquine, ammonium chloride and Palmostatin B alone did not significantly increase membrane recruitment of endogenous Ykt6 (Fig. 5F,G). Inhibiting both endosomal acidification and depalmitoylation together significantly increased Ykt6 detection in the membrane fraction (Fig. 5F,G). Next, we checked whether Ykt6 was able to detach from membranes after Bafilomycin A1 release in the presence or absence of Palmostatin B. Bafilomycin-dependent Ykt6 attachment to membranes was reversible only in the absence of Palmostatin B, demonstrating that depalmitoylation is the final step of membrane release (Fig. 5H,I). As Ykt6-3E remains palmitoylated and cannot detach from bound membranes anymore, a functional SNARE domain is required for the turnover of palmitoylation and regulation of membrane detachment.

To confirm the functional role of the Ykt6 SNARE domain *in vivo*, we mutated four serine residues to alanine (Ykt6-4A) or glutamic acid (Ykt6-4E) within the SNARE layers of *Drosophila* Ykt6 (Fig. S5A). Prolonged knockdown or permanent loss of Ykt6 is cell lethal, probably owing to lysosomal dysfunction (Matsui et al., 2018). Time-controlled RNAi of Ykt6 in the posterior compartment of WIDs caused intracellular Wg accumulation compared with the anterior control compartment (Fig. 5J,K). Ykt6 knockdown also suppressed Wnt target gene expression (Fig. S6A) and ultimately led to wing notches in adult flies, indicating blocked Wg secretion and consequently Wnt signalling defects (Fig. 5L; Strigini and Cohen, 2000). The mutated SNARE constructs (Ykt6-4A or Ykt6-4E) and Ykt6-WT were expressed using enGal4 in addition to tubGAL80-mediated time-controlled RNAi of Ykt6 in the posterior WID. Inhibition of Wg secretion and wing notches were rescued by expression of Ykt6-WT and Ykt6-4A (Fig. 5M-O, left and middle panels, Fig. S6B), thus confirming its specificity. However, co-expression of Ykt6-4E resulted in Wg accumulation and adult wing defects (Fig. 5M-O right panel, Fig. S6B). Along these lines, Ykt6-WT and, to some extent, -4A, but not -4E, were able to rescue overall lethality of the mutant alleles *ykt6^A^* and *ykt6^C^* (Fig. S6C). This indicates that Ykt6 requires a functional SNARE domain for both normal cellular growth and Wg secretion *in vivo*.

### Ykt6 recycles Wg via Rab4 endosomes

**Fig. 6.**
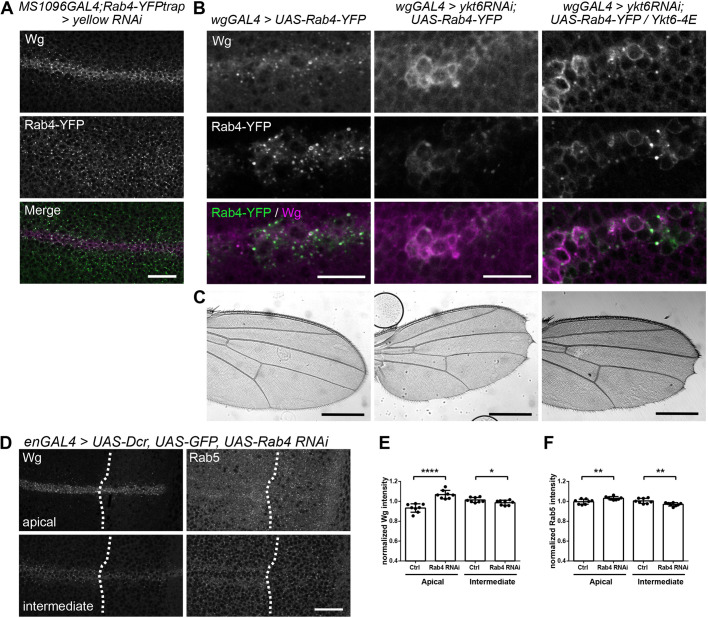
**Ykt6 recycles Wg via Rab4 endosomes.** (A) Yellow RNAi was expressed with MS1096GAL4 in the wing pouch in an endogenously tagged Rab4-YFP background. Maximum intensity projection of three sections (distance 0.5 µm) depicted for visualization. Scale bar: 20 µm. (B) UAS-Rab4-YFP was expressed with wgGAL4 alone, in combination with ykt6 RNAi or ykt6 RNAi and Ykt6-4E. A single subapical section is depicted. Scale bars: 10 µm. (C) Adult wings of the crosses from B to show adult wing notches. Scale bars: 500 µm. These wings are representative of more than 10 wings from three independent experiments. (D) RNAi against Rab4 was expressed with enGAL4, UAS-GFP,UAS-Dcr and stained for Wg and Rab5. Maximum intensity projection of three apical (upper panels) and intermediate (lower panels) sections is depicted for visualization. Scale bar: 20 μm. (E) Quantification of Wg apical versus intermediate fluorescence intensity in *n*=8 biologically independent samples from D. Data are mean±s.d., ****P<0.0001, *P=0.04. (F) Quantification of Rab5 apical versus intermediate fluorescence intensity in *n*=8 biologically independent samples from D. Data are mean±s.d., **P=0.006.

Based on our findings that Ykt6 is recruited to membranes by endosomal deacidification and genetically interacts with Hrs, we investigated whether Ykt6 recycles Wg from sorting endosomes to the apical surface for secondary, long-range secretion, possibly on cytonemes or extracellular vesicles (Gross et al., 2012; Stanganello et al., 2015). In mammalian cells, Rab4 directs fast recycling from early endosomes to the plasma membrane, whereas Rab11 mediates a slow recycling route from MVBs towards the plasma membrane (De Renzis et al., 2002). In WIDs, localization of the slow recycling endogenously tagged Rab11-YFP, as well as UAS-Rab11-YFP, were not affected upon Ykt6 knockdown (Fig. S6D,E), supporting the previous finding that Ykt6 does not influence MVB biogenesis (Fig. 4C,D). We next analysed the possibility that Wg might be recycled via a fast Rab4-dependent way. Both an endogenously tagged Rab4-YFP and overexpressed UAS-Rab4-YFP partially colocalize with Wg in puncta under control conditions (Fig. 6A,B). In contrast, both Wg and UAS-Rab4-YFP accumulate together intracellularly at the plasma membrane in Ykt6 RNAi, indicating that Ykt6 mediates Wg trafficking via Rab4 recycling endosomes (Fig. 6B,C, left and middle panels). Co-expression of Ykt6-4E results in Wg accumulating together with Rab4 and does not rescue wing notches induced by impaired Wg secretion (Fig. 6B,C, right panel). In agreement with this recycling route, in RNAi of Rab4, Wg accumulated towards the apical membrane (Fig. 6D,E), similar to Rab5 (Fig. 6D,F). Taken together, our results position Ykt6 function at the level of sorting endosomes, upstream of MVB sorting and ILV formation. Ykt6 cytosol-to-membrane cycling is required in a Rab4 endosomal trafficking step to ensure proper extracellular Wnt levels for Wnt target gene activation.

## DISCUSSION

In this study, we have shown that Ykt6 recycles Wg to the membrane via Rab4-positive endosomes to regulate Wnt trafficking in the polarized wing epithelium of *Drosophila*. Counterintuitively, an essential step of this trafficking is Wg endocytosis from the apical membrane before final secondary secretion and subsequent Wnt signal activation. In particular, the SNARE domain of Ykt6 is required for cycling between cytosol and membranes*,* as Wg/Wnts are trafficked through the secretory pathway. Our results explain how post-endocytic Wnt trafficking and Ykt6 as a valve contribute to adjusting extracellular Wnt levels and proper gradient formation.

### Endosomal regulation of Wnt signalling

Early endosomes are a major sorting hub and crossroad for internalized receptors, cargo and membranes (reviewed by Jovic et al., 2010). Interestingly, three Wnt signalling processes converge in and separate from acidified endosomes: (1) separation of Wnt from its trafficking receptor Evi and recycling of Evi via Retromer; (2) Wnt receptor activation; and (3) as we show here, secondary secretion of Wnts. The pool of apically presented and subsequently endocytosed Wg might serve as a signalling reservoir that can be rapidly mobilized by Ykt6-mediated recycling to the membrane. A possible reason for this might be that endosomes sense Wnt signalling levels and fine-tune further Wnt secretion accordingly. In line with this idea, Wg is endocytosed apically, while its receptor Fz2 is internalized from the basolateral side and both meet in acidified endosomes for signal transduction (Hemalatha et al., 2016). Similarly, acidification by V-ATPase activity is required for Wnt receptor activation in vertebrates (Cruciat et al., 2010). MVBs are also important regulatory hubs for non-transcriptional Wnt signalling readout (Acebron et al., 2014; Albrecht et al., 2018; Taelman et al., 2010). We found that, upon Ykt6 depletion, extracellular Wnt levels are reduced, but Evi levels are unchanged. Thus, we excluded a role for Ykt6 in the passage of Evi/Wnt complexes to acidified endosomes and reasoned that Ykt6 is required for an Evi-independent step of Wnt trafficking. We thus propose a model in which Ykt6 is recruited to de-acidified endosomes to re-secrete more Wnts, ensuring proper receptor activation in a feedback loop.

Our finding that Ykt6 acts at the level of early endosomes suggests that it only affects exosomal Wnt sorting indirectly. Ykt6 depletion increases Hrs-positive but reduces FYVE-GFP-positive endosomes and no changes were observed in MVB morphology in WIDs (Fig. 4C,D). This is in line with our previous observation in human cells that Ykt6 depletion affects exosomal CD63 MVB sorting rather than their formation (Gross et al., 2012). Interestingly, Wg endocytosis from the apical side depends on HSPGs (Baeg et al., 2001; Perrimon and Lin, 1999; Selleck et al., 1999), which are also involved in cargo sorting onto exosomes via Alix and Syntenin (Ghossoub et al., 2014).

Our results on Ykt6 membrane recruitment and Rab4 recycling in *Drosophila* indicate that there is an additional level at which Wnt secretion is fine-tuned in the late secretory pathway by Ykt6 cytosol-to-membrane cycling. Wnts and other lipid-modified signalling molecules, such as Hedgehog (Hh), have a common mechanism of intracellular trafficking and secretion (reviewed by Brunt and Scholpp, 2018). In agreement with our findings, two different routes of secretion from WIDs have been proposed for Hh: secretion from: (1) the basolateral membrane on cytonemes/EVs (Bischoff et al., 2013; Gradilla et al., 2014); and (2) from the apical membrane after passage through Rab4 endosomes (D'Angelo et al., 2015). Whether an Ykt6-mediated fusion step via Rab4 is regulating Hh secretion remains to be investigated.

### SNARE Ykt6 in endosomal trafficking

A fundamental question in intracellular trafficking is how specificity and directionality can be achieved. Peripheral membrane proteins have an advantage over transmembrane proteins in that their subcellular localization can be rapidly modulated. We identified putative phosphorylation sites within the SNARE domain of Ykt6 that allow membrane recruitment and stabilization. This mechanism is required for Ykt6 membrane-to-cytosol cycling and its function in Wnt secretion. Our rescue experiments show that phosphomimicking mutations stabilize Ykt6 at membranes and fail to rescue Wnt trafficking via Rab4 recycling endosomes. In general, members of the SNARE family are regulated by post-translational modifications such as monoubiquitylation (Syx5) (Huang et al., 2016) or palmitoylation (SNAP25) (Gonzalo and Linder, 1998). A recent study described phosphorylation sites within the SNARE domain of non-neuronal SNAREs conserved over the plant, fungi and animal kingdoms (Malmersjö et al., 2016). As shown for VAMP8, mutation of these sites inhibits fusion of secretory granules (Malmersjö et al., 2016). Further work will be required to determine which phosphorylation sites of the Ykt6 SNARE domain are physiologically relevant and whether they precede and direct membrane binding or stabilize previous membrane attachment. As a proposed stress sensor in yeast (Dietrich et al., 2004), our results confirm that the majority of human Ykt6 localizes to the cytoplasm, potentially serving as a reserve pool to release trafficking stress at different levels and under specific circumstances.

Our observed trafficking direction towards the plasma membrane oppose recent studies, in which Ykt6 was implicated in non-canonical autophagosome formation under starvation conditions (Kimura et al., 2017; Matsui et al., 2018; Takáts et al., 2018). However, first, we investigated Ykt6 function under normal growth conditions, as Wnt secretion is strongly reduced under starvation (Mihara et al., 2016) and found accumulation of Wg and Rab4 close to the plasma membrane, in combination with an increase in early but a decrease in late endosomal markers. Second, we found a genetic interaction between Ykt6 and Hrs, as Hrs knockout rescued Ykt6 RNAi lethality. These results fit with a re-routing of endosomal trafficking towards the extracellular space. Indeed Matsui and colleagues have proposed that lysosomal dysfunction is the cause of Ykt6 lethality (Matsui et al., 2018). Another Longin SNARE, Sec22B, mediates unconventional secretion of cytosolic proteins via autophagosome fusion with the plasma membrane (Kimura et al., 2017). Ykt6 activation via its conformational switch in the SNARE domain raises the interesting possibility of integrating different upstream signalling pathways and determining local activation of Ykt6 and therefore direction of trafficking events. In line with our findings, Ykt6 was shown to increase leucine and isoleucine uptake under starvation conditions by increasing the surface level of their transporters (Saito et al., 2019). Our BioID data confirm that Ykt6 acts proximal to very different cellular processes, such as endocytosis, RNA transport and metabolic signalling pathways, which could contribute to its RNAi-induced cell growth defects. It remains to be determined whether Ykt6 activation is a directional switch in endosomal trafficking towards the plasma membrane or lysosomal degradation.

Taken together, we have shown that Ykt6 cytosol-to-membrane cycling is required for Wnt secretion from endosomes. With its ability to adapt to multiple cellular localizations, Ykt6 is an ideal candidate for orchestrating selected cargo recycling of secreted morphogens such as Wnt, in the endosomal system. Further investigation is required to understand the regulatory networks upstream of Ykt6 endosomal trafficking at the crossroad of secretion and degradation.

## MATERIALS AND METHODS

### Plasmids and siRNA

The coding region of *Drosophila* Ykt6 was amplified and the PCR product recombined into pDONR221 vector using the Gateway BP Clonase II Enzyme mix (Life Technologies). Point mutations of potential phosphorylation sites (S175, S182, T188 and T192) were introduced by site-directed mutagenesis. For generation of transgenic flies, constructs were subcloned into expression vectors pUASt-attB-rfA-mCherry (a kind gift from Sven Bogdan, Philipps University Marburg, Germany) by LR recombination (Life Technologies). Human Ykt6 was amplified from hYkt6-Myc [C-terminal myc-destination plasmids (DKFZ – Genomics and Proteomics Core Facility)] and the PCR product inserted into pcDNA3.1MycBioID (Addgene 35700). Point mutations for Ykt6-3A (S174A, T181A and S187A), Ykt6-3E (S174E, T187E and S181E), F42A, C194A, C195A and relevant combinations were introduced by site-directed mutagenesis. The MycBioID tag was removed using NheI/XhoI to obtain untagged constructs in pcDNA3.1. The following expression constructs were used: TCF4/Wnt- Firefly Luciferase (Demir et al., 2013), Actin-Renilla Luciferase (Nickles et al., 2012), pCMV-Wnt3A (Gross et al., 2012) and DsRed-Rab5-QL (E. De Robertis, University of California, Los Angeles, USA; Addgene 29688). Dharmacon siRNA SMARTpools are listed in Table S3.

### Antibodies

Antibodies were used against Calnexin [1:1000 western blot (WB); rabbit (sc-11397), Santa Cruz); 1:10 immunofluorescence (IF); mouse (Cnx99A 6-2-1), DSHB], CD81 (1.3.3.22) [1:1000 WB; mouse (DLN-09707), Dianova], GAPDH (6C5) [1:5000 WB; mouse (AM4300), Ambion], GFP [1:1000 IF; mouse (A11120) and rabbit (A11122), Molecular Probes], GM130 [1:300 IF; mouse (610823), BD], GM130 [1:500 IF; rabbit (ab30637) Abcam], Hrs [1:10 IF; mouse (Hrs8-2 and Hrs27-4), DSHB], Hsc70 [1:2000 WB; mouse (sc-7298), Santa Cruz], Lamp1 [1:100 IF; rabbit (ab30687), Abcam], mCherry [1:1000 IF; rabbit (ab167453), Abcam], Rab5 [1:500; rabbit (ab31261), Abcam], Rab7 [1:10; mouse (Rab7), DSHB], Sec22, Syb and Vamp7 [IF; 1:250, kind gifts from Andrew A. Peden, The University of Sheffield, UK (Gordon et al., 2017)], Sens [IF; rabbit; 1:1000, a kind gift from Hugo Bellen, Baylor College of Medicine, Houston, TX, USA (Nolo et al., 2000)], Syx1A [1:10; mouse (8C3), DSHB], TSG101 [1:1000 WB; rabbit (HPA006161), Sigma], Wg [1:3 for extracellular and 1:20 for total staining (mouse, 4D4, DSHB)], Wnt3A [1:500 WB; rabbit, Abcam, ab172612), Wnt5A [1:500 WB; rabbit (2530), CST], Evi/Wls [1:500 IF; rabbit, a kind gift from Konrad Basler, University of Zurich, Switzerland), and Ykt6 [1:1000 WB and IF; mouse (sc-365732), Santa Cruz]. Secondary antibodies directed against the species of interest were coupled to Alexa Fluor 488, 568, 594 and 647 (IF, 1:500, Invitrogen), and 680RD and 800CW (WB, 1:20,000, LiCor).

### *Drosophila* stocks and genetics

The following *Drosophila* stocks were used in this study: *en-GAL4, UAS-GFP* (chr. II, a gift from J. Grosshans, Philipps University Marburg, Germany), *wg-Gal4* (chr. II, a gift from S. Cohen, University of Copenhagen, Denmark) and *UAS-GFP-Myc-2XFYVE* (chr. III, a gift from M. Gonzalez-Gaitan, University of Geneva, Switzerland). The following stocks were obtained from Bloomington Drosophila Stock Center: *da-GAL4* (5460), *UAS-Dcr; enGAL4,UAS-GFP* (25752), *tub-GAL80TS* (7108), MS1096-GAL4 (8860), *ykt6^A^FRT19A/FM7c,Kr-GAL4,UAS-GFP* (57143), *ykt6^C^FRT19A/FM7c,Kr-GAL4,UAS-GFP* (57142), *His2Av-GFP,hsFlp,FRT19A* (32045), FRT19A (1709), *vas-PhiC31; attP.ZH-86Fb* (24749), AliX TRiP (33417), Hrs TRiP (28026 and 33900), *UAS-Rab4-YFP* (9767), *UAS-Rab5-YFP* (24616), *UAS-Rab5Q88L-YFP* (9773), UAS-Rab11-YFP (50782), Rab4-YFP trap (62542) and Rab11-YFP trap (62549). The following UAS-RNAi stocks were obtained from Vienna Drosophila RNAi Center: ALiX (GD32047), AP-2α (GD15565), Evi (GD5214 and KK 103812), Rab4 (KK106651), Sec22 (KK100766), Snx3 (KK104494), Syb (KK102922) and Ykt6 (KK105648). Additional RNAi lines used for the screens in Fig. 3A are listed in Table S1. UAS-Ykt6 transgenic lines were generated according to standard protocols by φC31 integrase-mediated site-specific insertion in the attP landing site at ZH-86Fb (Bischof et al., 2007). We sequenced the *ykt6* mutant allele stocks and realized that the annotation at FlyBase/Bloomington is not correct: *ykt6^A^* (BL57143), annotated as M1I in fact carries Q62R, the mutation in the Longin domain. *ykt6^C^* (BL57142), annotated as Q62R in fact carries M1I, the mutation in the start codon.

Fly stocks were kept on standard medium containing agar, yeast and corn flour. Crosses were performed at 25°C except for *tub–Gal80TS* crosses, which were moved to 29°C 3 days before dissection of wing imaginal discs. To generate negatively marked *ykt6* mutant and *FRT* control clones in the wing imaginal disc under the control of *hsFlp*, animals of the appropriate genotype were heat-shocked 4 days after egg laying for 2 h at 37°C on 2 consecutive days and dissected on the next day at the wandering L3 stage.

### Cell culture and transfection

Hek293T, HCT116 and SkBr3 cells were maintained in DMEM (Gibco) supplemented with 10% fetal calf serum (Biochrom) at 37°C in a humidified atmosphere with 5% CO_2_. Cells were transiently transfected with Screenfect siRNA for siRNA and Screenfect A (Screenfect) for plasmids according to the manufacturer's instructions and checked regularly for mycoplasma contamination and authenticated.

### Cell fractionation

Cells were fractionated as described previously (Baghirova et al., 2015), briefly HEK293T cells were seeded and transfected with Ykt6-WT plasmid. At 48 h post-transfection, cells were lysed on ice with 1 ml of Lysis buffer A (150 mM NaCl, 50 mM Hepes, 0.1% saponin, 1 M glycerol and 1% PIC), then centrifuged at 2000 ***g*** for 10 min at 4°C and the supernatant (cytosolic fraction) was transferred to a new tube. The pellet was lysed in 1 ml of Lysis Buffer B (150 mM NaCl, 50 mM Hepes, 1% Igepal, 1 M glycerol and 1% PIC) and incubated by rotating for 30 min at 4°C. It was then centrifuged at 7000 ***g*** for 10 min at 4°C and the supernatant transferred to a new tube (membrane fraction). Proteins in both fractions were precipitated with methanol/chloroform and water as described previously (Wessel and Flügge, 1984).

### Blue sepharose precipitation

The relative amount of Wnts secreted into cell culture supernatant was analysed using blue sepharose precipitation as described previously (Glaeser et al., 2016; Willert et al., 2003). Briefly, cells were transiently transfected in 6-well plates with 1 µg of Wnt3A plasmids. At 72 h after transfection the supernatant was collected and centrifuged at 1500 ***g*** to remove cell debris, transferred to a fresh tube and rotated at 4°C for 1 h with 1% Triton X-100 and 40 µl of blue sepharose beads. The samples were washed and eluted from the beads using 2×SDS buffer with β-mercaptoethanol and analysed by immunoblotting.

### Immunostaining, microscopy and image analysis

For IF, cells were reverse transfected with siRNAs, seeded in 6-well dishes or on 8-well microscopic coverslips, transfected 24 h later with indicated plasmids and fixed with 4% paraformaldehyde 48–72 h later. Cells were permeabilized with 0.1% Triton X-100 and blocked in 10% BSA/PBS. Primary antibodies in PBS were incubated for 1 h at room temperature and antibody binding visualized using fluorochrome-conjugated secondary antibodies.

Immunostaining of wing imaginal discs was performed as per standard procedures. Total and extracellular Wg staining were carried out as previously described (Strigini and Cohen, 2000). Staining and microscopy conditions were kept identical for discs used for comparisons. Imaginal discs were mounted in Mowiol and images were taken using a Zeiss LSM780 confocal microscope. *Z* stacks were generated with 0.5-1 µm intervals using a Plan Neofluar 63×/oil NA 1.4 objective. Confocal images were processed with Zen lite (Zeiss), Fiji/ImageJ (NIH) (Rueden et al., 2017; Schindelin et al., 2012; Schneider et al., 2012) and Affinity Designer (Affinity). Quantification of colocalization was performed by calculating Pearson's coefficients of *z*-stacks using the Fiji/ImageJ PlugIn JaCoP (Bolte and Cordelières, 2006). Rab5Q88L and FYVE endosome sizes were quantified manually using Fiji/ImageJ. Details on image analysis are provided in the supplementary Materials and Methods.

### Wg endocytosis assay

To monitor Wg endocytosis and intracellular trafficking, WIDs were incubated in mouse anti-Wg (1:5, 4D4, DSHB) for 1 h at 22°C. To remove extracellular antibody signal, WIDs were rinsed three times in PBS and acid washed in 0.1 M glycine-HCl buffer (pH 3.5) for 30 s at room temperature. WIDs were rinsed three more times in PBS before being fixed and stained as described previously (Strigini and Cohen, 2000).

### Electron microscopy

Wing imaginal discs were fixed in 2.5% glutaraldehyde in 100 mM phosphate buffer (pH 7.2), washed in 100 mM phosphate buffer and postfixed in 2% osminum tetroxide in phosphate buffer for 1 h on ice. After contrasting en bloc in 2% uranyl acetate, the specimens were dehydrated in ethanol and embedded in araldite using acetone as an intermediate solvent. Thin sections were stained with 2% uranyl acetate and lead citrate. Sections were observed under an EM 109 (Zeiss) microscope at 80 KV. Quantification of MVB diameter was carried out manually in Fiji/ImageJ (NIH) (Rueden et al., 2017; Schindelin et al., 2012; Schneider et al., 2012).

### Click palmitoylation assay

Click assay was performed as described previously (Haberkant et al., 2016). In short, HEK293T cells were seeded, then transfected with plasmids (YKT6-WT BioID, YKT6-3E BioID and Wnt3A) in DMEM supplemented with 10% FBS. ω-Alkynyl palmitic acid (Alk-C16) was dissolved in ethanol to a final concentration of 50 mM and stored at −80°C. Alk-C16 was diluted to a final concentration of 100 μM in DMEM supplemented with 5% FBS (fatty acid-free) sonicated for 15 min at room temperature in a water bath and then allowed to precomplex for another 15 min. Alk-C16-containing medium was added to cells and partially replaced after 24 h. At 72 h post-transfection, cells were lysed (PBS with 1% Triton x-100, 0.1% SDS, PIC), then centrifuged at 16,000 ***g*** for 5 min at 4°C. Lysates were then precipitated with Wessel-Flugge Protein precipitation. The click labelling reaction [0.1 mM biotin-azide, 1 mM Tris(2-carboxyethyl)phosphine hydrochloride (TCEP, Sigma-Aldrich) dissolved in water, 0.1 mM Tris[(1-benzyl-1H-1,2,3-triazol-4-yl)methyl]amine (TBTA, Sigma–Aldrich) dissolved in DMSO and 1 mM CuSO4 in water] was incubated by shaking for 2 h at 37°C under dark conditions. After the click reaction, the samples were precipitated with 10× methanol overnight at −80°C, then centrifuged and washed again with ice-cold methanol, The dried pellet was resuspended in 4% SDS. Click-biotinylated proteins precipitated with High Capacity Neutravidin Agarose Resin (Thermo Scientific). Samples were washed with 1% SDS and eluted, then analysed further by immunoblotting.

### BioID pull down and mass spectrometry

For large-scale BioID pull down, Hek293T cells were seeded and 24 h later transfected with BioID-WT or mock constructs. At 36 h post-transfection 50 μM biotin was added overnight. Cells were washed with PBS twice and harvested in Ripa Lysis buffer [50 mM Tris-HCl (pH 7.5), 150 mM NaCl, 1% Igepal, 0.5% sodium desoxycholate, 0.1% SDS] containing 1× Complete protease inhibitor (Life Technologies). After centrifugation at 16,500 ***g*** for 10 min, lysates were boiled for 5 min in non-reducing SDS sample buffer [300 mM Tris-HCl (pH 6.8), 12% SDS, 0.05% bromophenol blue, 60% glycerol, 12 mM EDTA], either fully separated or run short-distance (1.5 cm) on a 4-12% NuPAGE Novex Bis-Tris Minigel (Invitrogen). Gels were stained with Coomassie Blue for visualization purposes. Full lanes were sliced into 23 equidistant slices regardless of staining, short runs were cut out as a whole and diced. After washing, gel slices were reduced with dithiothreitol (DTT), alkylated with 2-iodoacetamide and digested with trypsin overnight. The resulting peptide mixtures were then extracted, dried in a SpeedVac, reconstituted in 2% acetonitrile/0.1% formic acid/ (v:v) and prepared for nanoLC-MS/MS as described previously (Atanassov and Urlaub, 2013).

For generation of a peptide library for SWATH-MS, equal aliquots from each sample were pooled to a total amount of 80 µg and separated into eight fractions using a reversed-phase spin column (Pierce High pH Reversed-Phase Peptide Fractionation Kit, Thermo Fisher Scientific). Mass spectrometry analysis protein digests were separated by nanoflow chromatography. Either 25% of gel slices or 1 µg aliquots of digested protein were enriched on a self-packed precolumn (0.15 mm ID×20 mm, Reprosil-Pur120 C18-AQ 5 µm, Dr Maisch, Ammerbuch-Entringen, Germany) and separated on an analytical RP-C18 column (0.075 mm ID×250 mm, Reprosil-Pur 120 C18-AQ, 3 µm, Dr Maisch) using a 30 to 90 min linear gradient of 5-35% acetonitrile/0.1% formic acid (v:v) at 300 nl min^−1^.

For spectral counting analysis, the eluent was analysed on a Q Exactive hybrid quadrupole/orbitrap mass spectrometer (ThermoFisher Scientific) equipped with a FlexIon nanoSpray source and operated under Excalibur 2.4 software using a data-dependent acquisition method. Each experimental cycle was of the following form: one full MS scan across the 350-1600 m/z range was acquired at a resolution setting of 70,000 FWHM, an AGC target of 1×10e6 and a maximum fill time of 60 ms. Up to the 12 most abundant peptide precursors of charge states 2 to 5 above a 2×10e4 intensity threshold were then sequentially isolated at 2.0 FWHM isolation width and fragmented with nitrogen at a normalized collision energy setting of 25%. The resulting product ion spectra were recorded at a resolution setting of 17,500 FWHM, AGC target of 2×10e5 and a maximum fill time of 60 ms. Selected precursor m/z values were then excluded for the following 15 s. Two technical replicates per sample were acquired.

SWATH-MS library generation was performed on a hybrid triple quadrupole-TOF mass spectrometer (TripleTOF 5600+) equipped with a Nanospray III ion source (Ionspray Voltage 2400V, Interface Heater Temperature 150°C, Sheath Gas Setting 12) and controlled by Analyst TF 1.7.1 software build 1163 (all AB Sciex), using a Top30 data-dependent acquisition method with an MS survey scan of m/z 380–1250 accumulated for 250 ms at a resolution of 35,000 full width at half maximum (FWHM). MS/MS scans of m/z 180–1500 were accumulated for 100 ms at a resolution of 17,500 FWHM and a precursor isolation width of 0.7 FWHM, resulting in a total cycle time of 3.4 s. Precursors above a threshold MS intensity of 200 cps with charge states 2+, 3+ and 4+ were selected for MS/MS; the dynamic exclusion time was set to 15 s. MS/MS activation was achieved by CID using nitrogen as a collision gas and using the manufacturer's default rolling collision energy settings. Two technical replicates per reversed phase fraction were analysed to construct a spectral library.

For quantitative SWATH analysis, MS/MS data were acquired using 100 variable size windows (Zhang et al., 2015) across the 400-1200 m/z range. Fragments were produced using rolling collision energy settings for charge state 2+, and fragments acquired over an m/z range of 180-1500 for 40 ms per segment. Including a 250 ms survey scan, this resulted in an overall cycle time of 4.3 s. Two replicate injections were acquired for each biological sample.

### Mass spectrometry data processing

For spectral counting analysis, peaklists were extracted from the raw data using Raw2MSMS software v1.17 (Max Planck Institute for Biochemistry, Martinsried, Germany). Protein identification was achieved using MASCOT 2.5.1 software (Matrixscience, London, UK). Proteins were identified against the UniProtKB *Homo sapiens* reference proteome (revision 02-2017, 92,928 entries). The search was performed with trypsin as enzyme and iodoacetamide as cysteine blocking agent. Up to two missed tryptic cleavages and methionine oxidation as a variable modification were allowed for. Search tolerances were set to 10 ppm for the precursor mass and 0.05 Da for fragment masses. Scaffold software version 4.4.1.1 (Proteome Software) was used to validate MS/MS-based peptide and protein identifications. Protein and peptide identifications were filtered to 1% FDR using a concatenated forward-and-reverse decoy database approach. Relative quantification of proteins in the samples was achieved by two-sided *t*-tests of normalized spectral counts using a Benjamini-Hochberg-corrected *P* value of 0.05 to judge significance. To allow for the calculation of low abundance protein ratios, a minimum value of three spectral counts was introduced where necessary to avoid division by zero issues.

For SWATH-MS analysis, protein identiﬁcation was achieved using ProteinPilot Software version 5.0 build 4769 (AB Sciex) at ‘thorough’ settings. MS/MS spectra from the combined qualitative analyses were searched against the UniProtKB *Homo sapiens* reference proteome (revision 02-2017, 92,928 entries) augmented with a set of 51 known common laboratory contaminants to identify 597 proteins at a false discovery rate (FDR) of 1%. Spectral library generation and SWATH peak extraction were achieved in PeakView Software version 2.1 build 11041 (AB Sciex) using the SWATH quantitation microApp version 2.0 build 2003. Following retention time correction on endogenous peptides spanning the entire retention time range, peak areas were extracted using information from the MS/MS library at an FDR of 1% (Lambert et al., 2013). The resulting peak areas were summed to peptide and protein area values, which were used for further statistical analysis. Reactome functional network analysis (Gobert et al., 1996) was performed with Cytoscape (www.cytoscape.org) and Kegg pathway analysis was performed using David (Huang et al., 2009a,b).

### Immunoblot

To analyse total cell lysates using immunoblot, cells were lysed in SDS–PAGE sample buffer and boiled for 5 min. Proteins were separated on 4–12% gradient gels (Bolt Bis-Tris Plus Gels, ThermoFisher Scientific) and transferred to PVDF membrane (Merck). After blocking with 5% (wt/vol) milk-TBST, membranes were incubated with Licor-800nm–conjugated streptavidin (1:20,000, ab7403; Abcam) for 30 min. After detecting biotinylated proteins, membranes were subjected to detection with antibodies against Ykt6, cellular fraction markers already mentioned and Licor680nm–conjugated secondary antibodies.

### Ykt6 model prediction

A Ykt6-3E structural model was predicted using RaptorX (Källberg et al., 2012) and is based on the Ykt6 structure (3kyqA) as a template.

### Statistics

All experiments were carried out in at least biological triplicates. Error bars indicate s.d. Statistical significance was calculated by carrying out one-way ANOVA with Dunnett's multiple comparison test to compare a control mean with the other means or using an unpaired Student's *t*-test where appropriate. The data that support the findings of this study are available from the corresponding author upon reasonable request.

## Supplementary Material

Supplementary information

Reviewer comments
