# Supplementary material for: Ykt6-dependent endosomal recycling is required for Wnt secretion in the Drosophila wing epithelium
Source: Development. 2020 Aug 14;147(15):dev185421. doi: 10.1242/dev.185421 (PMC7438013; doi:10.1242/dev.185421)
Supplement: Supplementary information [file develop-147-185421-s1.pdf]

Supplementary information

Table S1: SNARE in vivo RNAi screening results.

Click here to Download Table S1

Table S2: Proteins identified by mass spectrometry from BioID control and Ykt6-WT samples in two biological replicates

Click here to Download Table S2

Table S3: Dharmacon siRNA SMARTpools

Gene Symbol	GENE ID	Gene Accession	GI Number	Sequence
siGENOME Non-targeting Control_5#				UGGUUUACAUGUCGACUAA
AP1S1	1174	NM_057089	148536832	AAUGGUACCUGGCCACUUC
AP1S1	1174	NM_057089	148536832	GCUCGAAAGCCCAAGAUGU
AP1S1	1174	NM_057089	148536832	CAUCGAGGGCCAAGACAAU
AP2S1	1175	NM_021575	70906431	AGACGAAGGUGCUGAAACA
AP2S1	1175	NM_021575	70906431	GGUCUUAAACGAAUAUUUC
AP2S1	1175	NM_021575	70906431	AACAGAAGCUGAUCGAGGA
ARPC1A	10552	NM_006409	22907051	ACGAAGUGCACAUCUAUAA
ARPC1A	10552	NM_006409	22907051	GAAUUAAUCGCGCAGCUAC
ARPC1A	10552	NM_006409	22907051	GUGGCACGAUGGCGAGGAA
ARPC1A	10552	NM_006409	22907051	GGAAGUGGAGCACGACUCA
CHMP2B	25978	NM_014043	40254865	GAAGAUGGCUGGAGCAAUG
CHMP2B	25978	NM_014043	40254865	UAAGGAAGCUUGCAAAGUU
CHMP2B	25978	NM_014043	40254865	GCUCGAAGCUUACCAUCUG
CHMP2B	25978	NM_014043	40254865	GCCAGGAUAUUGUGAAUCA
CTNNB1	1499	NM_001904	4503130	GCUGAAACAUGCAGUUGUA
CTNNB1	1499	NM_001904	4503130	GAUAAAGGCUACUGUUGGA
CTNNB1	1499	NM_001904	4503130	CCACUAAUGUCCAGCGUUU
DNM2	1785	NM_004945	56549118	CCGAAUCAAUCGCAUCUUC
DNM2	1785	NM_004945	56549118	GACAUGAUCCUGCAGUUCA
DNM2	1785	NM_004945	56549118	CCUCCGAGCUGGCGUCUAC
DNM2	1785	NM_004945	56549118	AGUCCUACAUCAACACGAA
GOSR2	9570	NM_054022	60499002	ACGAAUCACUGCAGUUUAA
GOSR2	9570	NM_054022	60499002	GAUCCAGUCUUGCAUGGGA
GOSR2	9570	NM_054022	60499002	CGAAAUCCAAGCAAGCAUA
GOSR2	9570	NM_054022	60499002	GAUAUUCAGCCGUCUAGAA
KIF5B	3799	NM_004521	4758647	GCAGUCAGGUCAAAGAAUA
KIF5B	3799	NM_004521	4758647	GAACUGGCAUGAUAGAUGA
KIF5B	3799	NM_004521	4758647	CAACAGACAUGUAGCAGUU
SEC22B	9554	NM_004892	94429049	GAAGAAGUGUUACAACGAG
SEC22B	9554	NM_004892	94429049	CUAAGCAACUCUUUCGAAA

SEC22B	9554	NM_004892	94429049	UAACAAUGAUCGCCCGAGU
SNAP25	6616	NM_003081	18765732	GCAAUGAGAUCGAUACACA
SNAP25	6616	NM_003081	18765732	GCGAAGGGCUGACCAGUUG
SNAP25	6616	NM_003081	18765732	GGAAAGCACCCGUCGUAUG
SNX1	6642	NM_148955	71772739	GGAAAGAGCUAGCGCUGAA
SNX1	6642	NM_148955	71772739	GAAAGGGACUUCGAGAGGA
SNX1	6642	NM_148955	71772739	GAAAAGAAGUGAUACGGUU
SNX1	6642	NM_148955	71772739	AGAACCACGUGAUCAAGUA
STX1A	6804	NM_004603	95147340	GGAACACGCGGUAGACUAU
STX1A	6804	NM_004603	95147340	GGAGGAGAUUCGAGGCUUC
STX1A	6804	NM_004603	95147340	ACAUAAAGAAGACAGCAAA
STX5	6811	NM_003164	94400931	GCAAGUCCCUCUUUGAUGA
STX5	6811	NM_003164	94400931	GAGCUAACAUAUAUCAUCA
STX5	6811	NM_003164	94400931	GAGCCCAGCUGGACGUUGA
STX7	8417	NM_003569	4507294	CAAAGAAACAGAUAAGUAC
STX7	8417	NM_003569	4507294	GCGAUUAUCAGUCUCAUCA
STX7	8417	NM_003569	4507294	GUCAAGGGCAGCAGAUUAU
STX7	8417	NM_003569	4507294	GAGUUUGUUGCUCGAGUAA
STX8	9482	NM_004853	296010812	CACCAAAGCUUACCGUGAC
STX8	9482	NM_004853	296010812	UCUUGUAACUCGAGAGAGA
STX8	9482	NM_004853	296010812	GAAUGAGGGUGCCGAACCA
STX8	9482	NM_004853	296010812	UGAGAUAAUUGACGACCUU
USE1	55850	NM_018467	154354977	CGUCGAGGCUGGAGCUAAA
USE1	55850	NM_018467	154354977	CUGAGGUGAUCAAUGAAUA
USE1	55850	NM_018467	154354977	GAGAUGGACGUAAGGAAGA
USE1	55850	NM_018467	154354977	CGAAUCAUGCCUAAACUCA
VAMP1	6843	NM_014231	40549444	CUCCUAACAUGACCAGUAA
VAMP1	6843	NM_014231	40549444	CAUCACAAUUUGAGAGCAG
VAMP1	6843	NM_014231	40549444	CCAUCAUCGUGGUAGUUAU
VAMP1	6843	NM_014231	40549444	AGGCACAAGUGGAGGAGGU
VAMP3	9341	NM_004781	42544205	GGCAGGCGCUUCUCAAUUU
VAMP3	9341	NM_004781	42544205	GGAUUACUGUUCUGGUUAU
VAMP3	9341	NM_004781	42544205	GCCAAGUUGAAGAGGAAAU
VAMP7	6845	NM_005638	27545446	GGAGAAAGAUUGGAAUUAU
VAMP7	6845	NM_005638	27545446	GUACUCACAUGGCAAUUAU
VAMP7	6845	NM_005638	27545446	AAGAAGAGGUUCCAGACUA
VTI1A	143187	NM_145206	113374155	CGUCCGACUUCGAAGGUUA
VTI1A	143187	NM_145206	113374155	CGUGAAAGACUUCGGGAAA
VTI1A	143187	NM_145206	113374155	CGAGGGAUGUACAGCAACA
VTI1A	143187	NM_145206	113374155	GGUCAGGAGAUGUUGGAAA
YKT6	10652	NM_006555	34304384	GCUCAAAGCCGCAUACGAU
YKT6	10652	NM_006555	34304384	GUGAGAAGCUAGAUGACUU
YKT6	10652	NM_006555	34304384	GAAGGUACUAGAUGAAUUC

Development • Supplementary information

Supplementary materials and methods

Image analysis

Fig. 3: exWg

MIP of 45 (Ykt6, distance 0,5 µm) or 25 (AP2, distance 1 µm) sections covering the entire stack depicted for visualization. For quantification the average intensity projection of the corresponding stack was used. The profile of the extracellular Wg staining in the shown ROI (1142 x 300 px^2, corresponding to 80x21 μm^2), comparing exWg in the anterior (control, no GFP) with the posterior (RNAi, GFP-positive) region for this one representative example, was plotted with Fiji. For quantification of several independent samples, two ROIs (500x500 px^2, corresponding to 1245 μm^2) were placed manually in the anterior (control) and posterior (RNAi) region of the corresponding stack. The mean fluorescence of five subapical sections (distance 1 µm) is measured with Fiji and averaged per stack. This corresponds to taking the mean fluorescence of an average intensity projection of this corresponding stack. Statistical significance was determined using Student's *t*-test (two-tailed) in GraphPad Prism 6 software.

Fig. 3: Evi

MIP of 15 apical sections (distance 1 µm) depicted for visualization. For quantification the average intensity projection of the corresponding stack was used. The profile of the Evi staining in the shown ROI (1428 x 300 px^2, corresponding to 100 x 21 μm^2), comparing Evi in the anterior (control, no GFP) with the posterior (RNAi, GFP-positive) region for this one representative example, was plotted with Fiji. For quantification of several independent samples, two ROIs (500x250 px^2, corresponding to 623 μm^2) were placed manually in the anterior (control) and posterior (RNAi) region of the corresponding stack. The mean fluorescence of five subapical sections (distance 1 µm) is measured with Fiji and averaged per stack. This corresponds to taking the mean fluorescence of an average intensity projection of this corresponding stack. For quantification of Evi punctae, a maximum intensity projection of the same five subapical sections as above (distance 1 µm) was generated and thresholded using Triangle algorithm implemented in Fiji. Two manually drawn ROIs were placed in the control and the RNAi region along the entire Evi expression domain. Particles > 4 px^2 were automatically counted using Fiji Particle Analyzer within the ROIs. The number of particles was normalized to the area quantified. Statistical significance was determined using Student's *t*-test (two-tailed) in GraphPad Prism 6 software.

Fig.4: Hrs

MIP of nine sections (distance 1 µm) for visualization. For quantification of several independent samples two ROIs (500x500 px^2, corresponding to 1245 μm^2) were placed manually in the anterior (control) and posterior (RNAi) region of the corresponding stack. The mean fluorescence of five subapical sections (distance 1 µm) is measured with Fiji and averaged per stack. This corresponds to taking the mean fluorescence of an average intensity projection of this corresponding stack. The sections to be measured were chosen based on nuclear staining. The nuclei had to be apparent in the entire section. Hrs staining is also very strong in the peripodial membrane, but this signal was not included. Statistical significance was determined using Student's *t*-test (two-tailed) in GraphPad Prism 6 software.

Development • Supplementary information

Fig.4: Lamp1
MIP of seven sections (distance 1 µm) are depicted for visualization. Quantification was done as for Hrs.

Fig.4: Rab5
MIP of 13 sections (distance 1 µm) are depicted for visualization. Quantification was done as for Hrs.

Fig.4: Rab7

MIP of seven sections (distance 1 µm) are depicted for visualization. Quantification was done as for Hrs.

Fig. 4: FYVE

For quantification of FYVE-labelled Wg punctae, a 30px XZ section within the Wg stripe was generated. In Fiji a ROI (1500 x 250 px^2, corresponding to 105 x 17.5 μm^2) was placed at the center of the Wg expression domain. Within this ROI, particles >6 px were automatically counted and colocalization determined using the Fiji Plugin ComDet.

Fig. 5: Wg in rescue crosses with Ykt6 SNARE-mutants
MIP of six sections (distance 0,5 µm) for visualization. For quantification of several independent samples a 5 µm average intensity projection of the corresponding stack was used. Two ROIs (100x300 px^2, corresponding to 145 μm^2) were placed manually in the anterior (control) and posterior (RNAi) region of the corresponding stack. The mean fluorescence is measured with Fiji. Statistical significance was determined using Student's *t*-test (two-tailed) in GraphPad Prism 6 software.

Development • Supplementary information

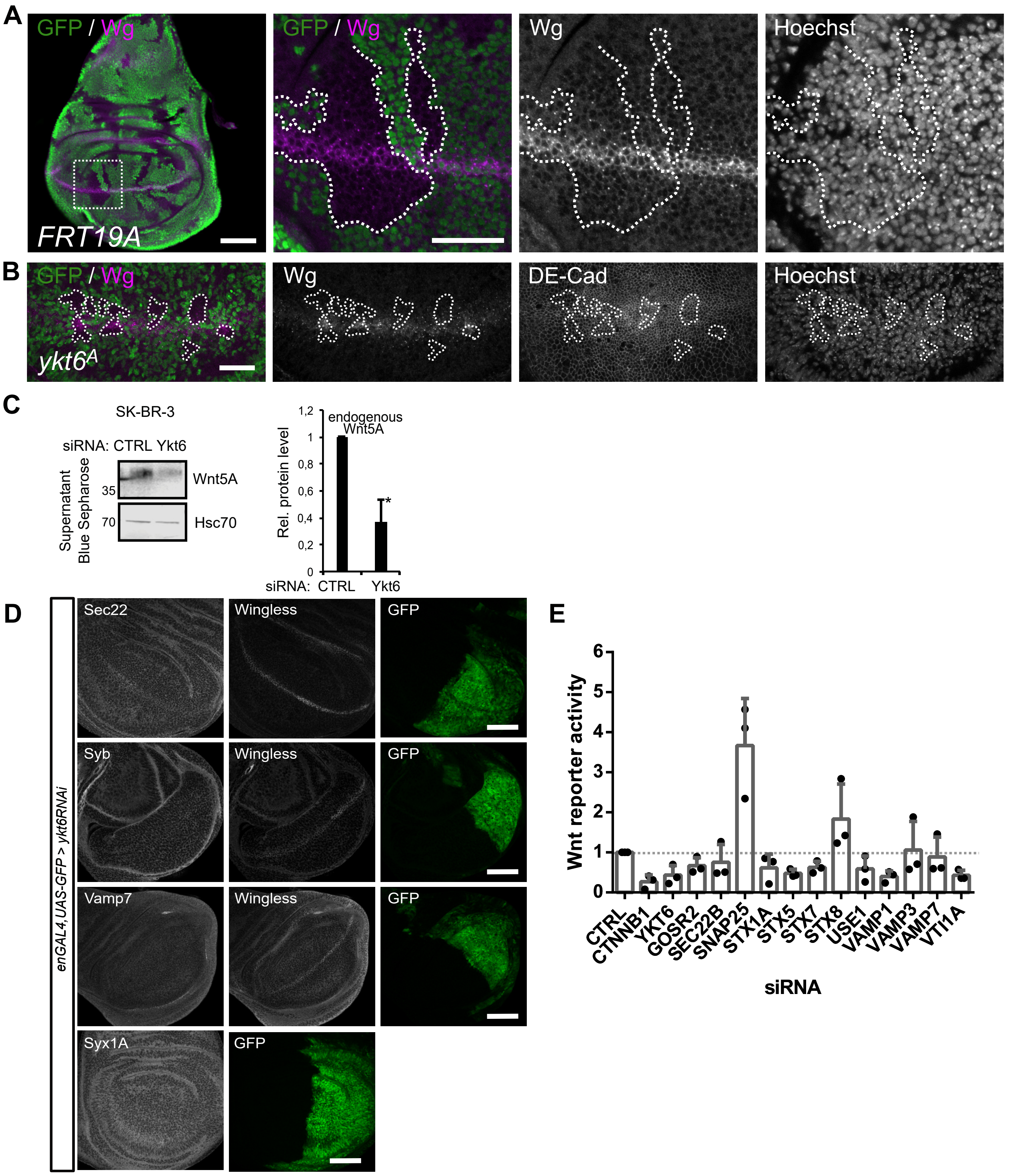

Fig. S1. Loss of Ykt6 blocks Wnt secretion.
(**A**) Induction of FRT19A control clones marked by the absence of GFP does not affect Wg secretion. The zoomed images to the right are enlarged views of the area marked with a dotted square in the overview image on the left. Maximum intensity projection of two subapical optical sections (distance 1 µm) are depicted for visualization. Scale bars, 50 µm in overview and 20 µm in other images. (**B**) Wingless protein accumulates in ykt6A clones, while DE-Cad staining is normal. Maximum intensity projection of three subapical (DE-Cad) and lateral (Wg and Hoechst) optical sections (distance 1 µm) are depicted for visualization. Scale bar 20 µm. (**C**) Wnt5A secretion from SkBr3 cells is reduced in Ykt6 knockdown cells. Quantification of three independent experiments, * p= 0,01 student t-test. (**D**) Knock-down of Ykt6 by RNAi in the posterior compartment of third instar WID marked by co-expression of GFP (engrailed-Gal4, UAS-GFP/UAS-ykt6RNAi) does not change the levels of Sec22, Syb, Vamp7 and Syx1A. Images are representative of >six WID per RNAi from two independent experiments. Scale bars represent 50 µm. (**E**) Wnt reporter assay of different SNAREs from three independent experiments. Not significant, one-way ANOVA.

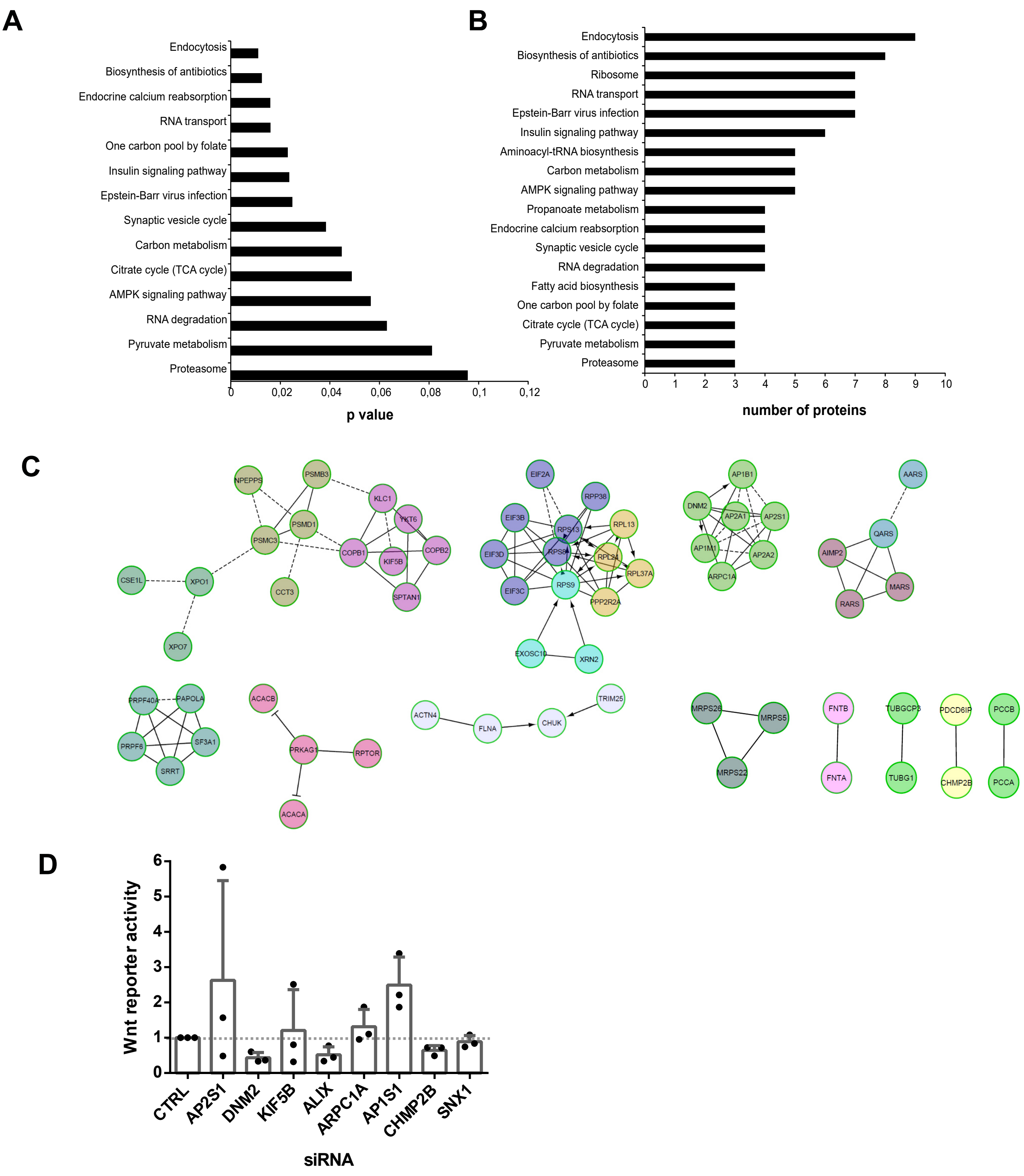

Fig. S2. Ykt6 acts on endosomal compartments after apical presentation.
(**A**) BioID-identified proteins were sorted by p-value for Kegg pathway enrichment in Ykt6-WT sample over control. (**B**) Number of proteins enriched in different Kegg pathways. (**C**) Reactome FI network analysis, networks with at least two nodes are displayed. (**D**) Wnt reporter assay of endocytosis pathway components from Fig. 3G from three independent experiments. Not significant, one-way ANOVA.

Development • Supplementary information

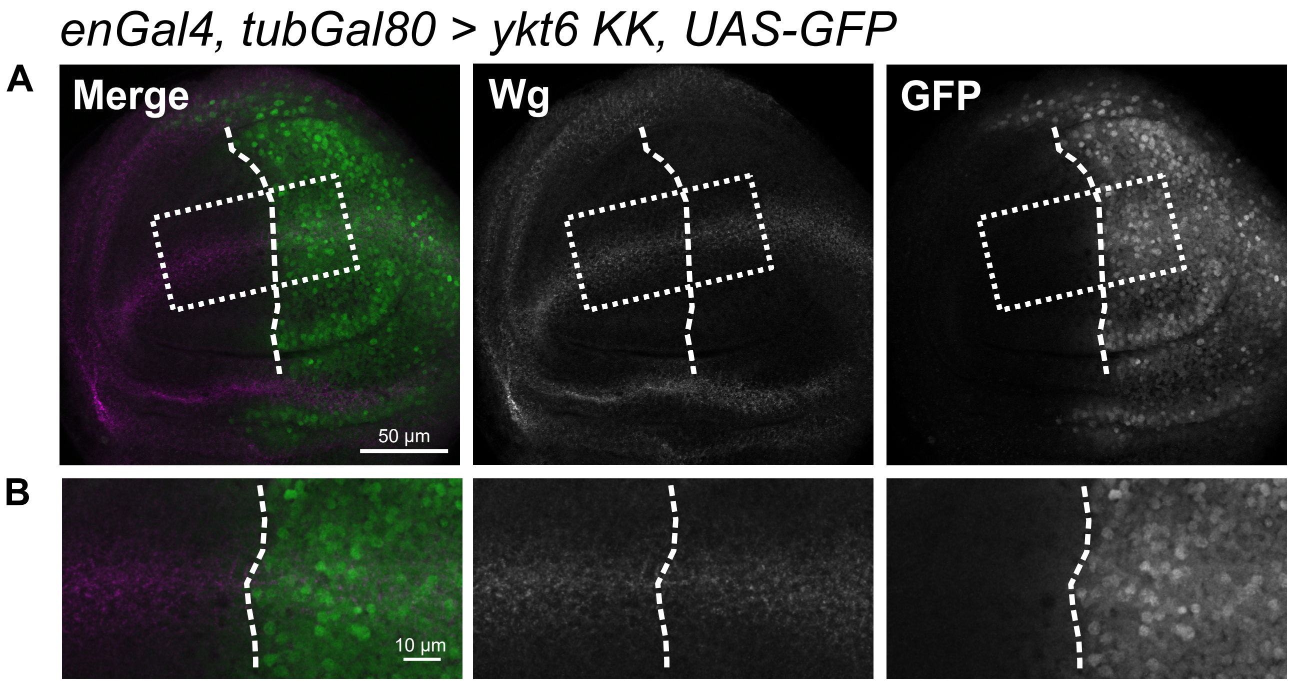

Fig. S3. Ykt6 knockdown is not sufficient to block Evi recycling.
(**A,B**) Endocytosis of anti-Wg antibody for 60min in enGal4-driven ykt6 knockdown WID. (**A**) Overview images. (**B**) Enlarged view of the area marked with a dotted square in (A). Scale bars as indicated.

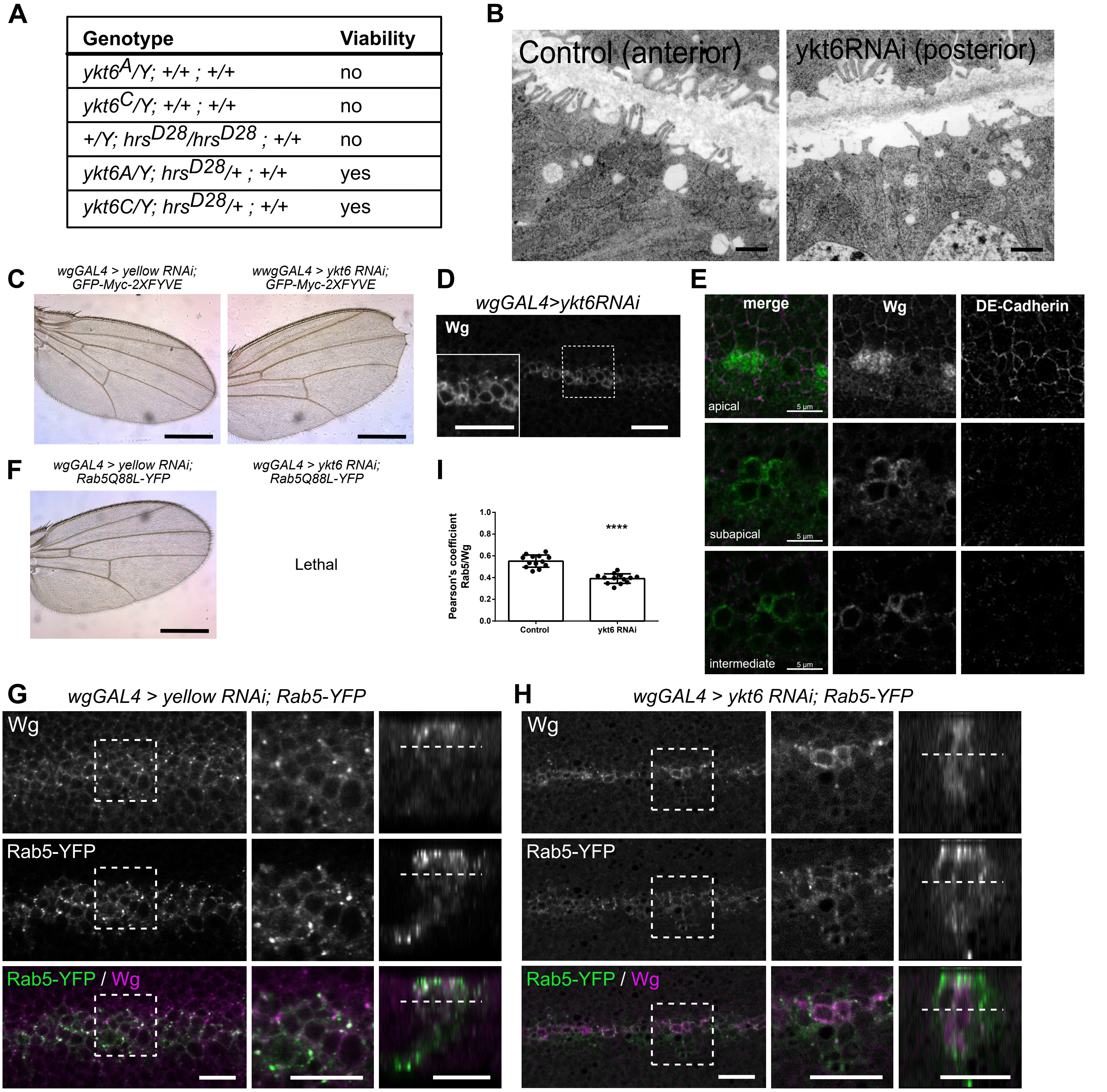

Fig. S4: Ykt6 acts on Wnt trafficking at the level of endosomes.
(**A**) Genetic interaction of *ykt6* and *hrs*. *ykt6* homozygous lethality can be rescued by removing one copy of *hrs*. (**B**) Electron microscopy images of apical membranes in WID of time-controlled RNAi of Ykt6 (engrailed-Gal4, UAS-GFP/UAS-ykt6RNAi; tubGal80-TS/+, larvae reared for three days at days at 29°C). Scale bar is 500 nm. (**C**) UAS-GFP-Myc-2xFYVE was expressed with wgGAL4 in combination with yellow (control, left panel) or ykt6 RNAi (right panel) to analyze for adult wing notches. Scale bars 500 µm. Representative of >10 wings from three independent experiments. (**D**) wgGAL4 mediated Ykt6 RNAi leads to cortical Wg accumulation. (**E**) High resolution images of Wg and DE-Cadherin localization in wgGAL4 mediated Ykt6 RNAi by Confocal Airy Scan imaging. (**F**) UAS-Rab5Q88L-YFP was expressed with wgGAL4 in combination with yellow (control, left panel) or ykt6 RNAi (right panel) to analyze for adult wing notches. Scale bars 500 µm. Representative of >10 wings from three independent experiments. (**G, H**) UAS-Rab5-YFP was expressed with wgGAL4 in combination with yellow (control) (G) or ykt6 RNAi (H). Images represent a single confocal section. Left panel depicts a cross section, middle panel a magnification of the region boxed in the left panel. The right panel is a transverse (YZ) section of the stack. The dashed line marks the corresponding focal plane of the images shown in the left and middle. Scale bars is 10 µm in all images. (**I**) Pearson's coefficient of Rab5/Wg is decreased upon ykt6 knockdown with enGAL4,UAS-GFP;tubGAL80TS.

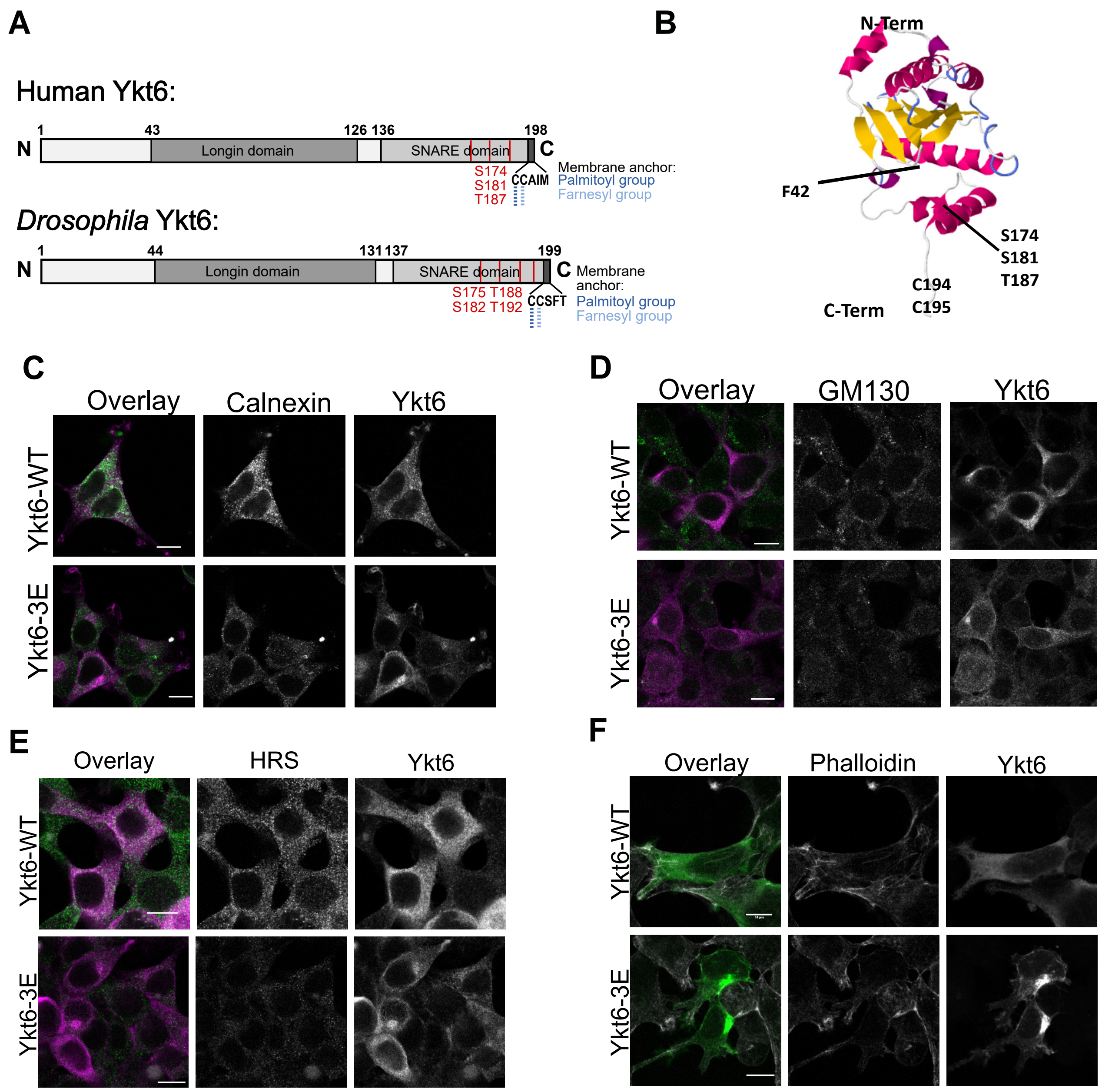

Fig. S5: Ykt6 SNARE domain is required for cycling between compartments. (**A**) Scheme of human and *Drosophila* Ykt6 with predicted phosphorylation sites in the SNARE domain. (**B**) Structural model of Ykt6 from template (3kyqA). Mutations as indicated: F42 in the Longin domain, S174, S181 and T187 in the SNARE domain and C194 and C195 in the CAAX motif for acylation. (**C-F**) Colocalization of Bio-ID-tagged Ykt6-WT and -3E in Hek293T cells with organelle markers for (**C**) ER (Calnexin), (**D**) Golgi (GM130), (**E**) endosomes (Hrs) and (**F**) F-actin (Phalloidin). Scale bars 10 µm and a representative field of view from three independent experiments is depicted.

Development • Supplementary information

A

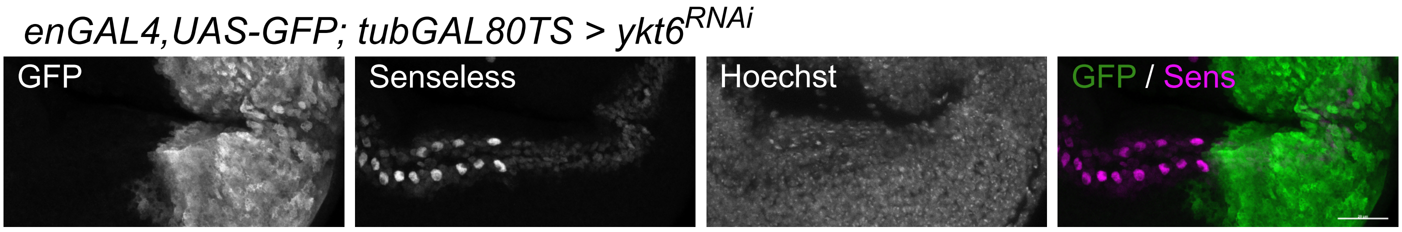

B

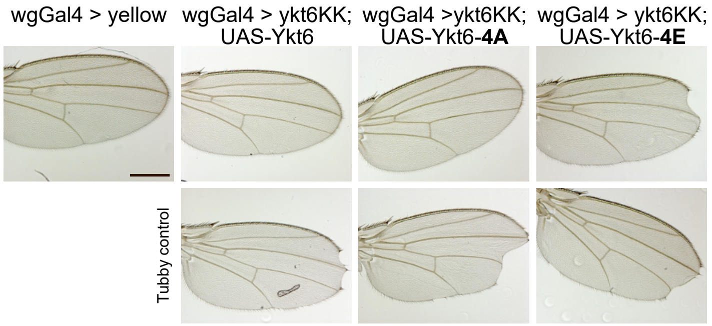

C

Genotype		Viability
ykt6^{A}/Y; ; daGAL4/Tsp42Ef-pTChW	**UAS control**	✓
ykt6^{A}/Y; ; daGAL4/Ykt6-pTWCh	**WT, untagged**	✓
ykt6^{A}/Y; ; daGAL4/Ykt6-**4A**-pTWCh2	**4A, untagged**	✓
ykt6^{A}/Y; ; daGAL4/Ykt6-**4E**-pTWCh5	**4E, untagged**	×
ykt6^{C}/Y; ; daGAL4/Tsp42Ef-pTChW	**UAS control**	✓
ykt6^{C}/Y; ; daGAL4/Ykt6-pTWCh	**WT, untagged**	✓
ykt6^{C}/Y; ; daGAL4/Ykt6-**4A**-pTWCh2	**4A, untagged**	×
ykt6^{C}/Y; ; daGAL4/Ykt6-**4E**-pTWCh5	**4E, untagged**	×

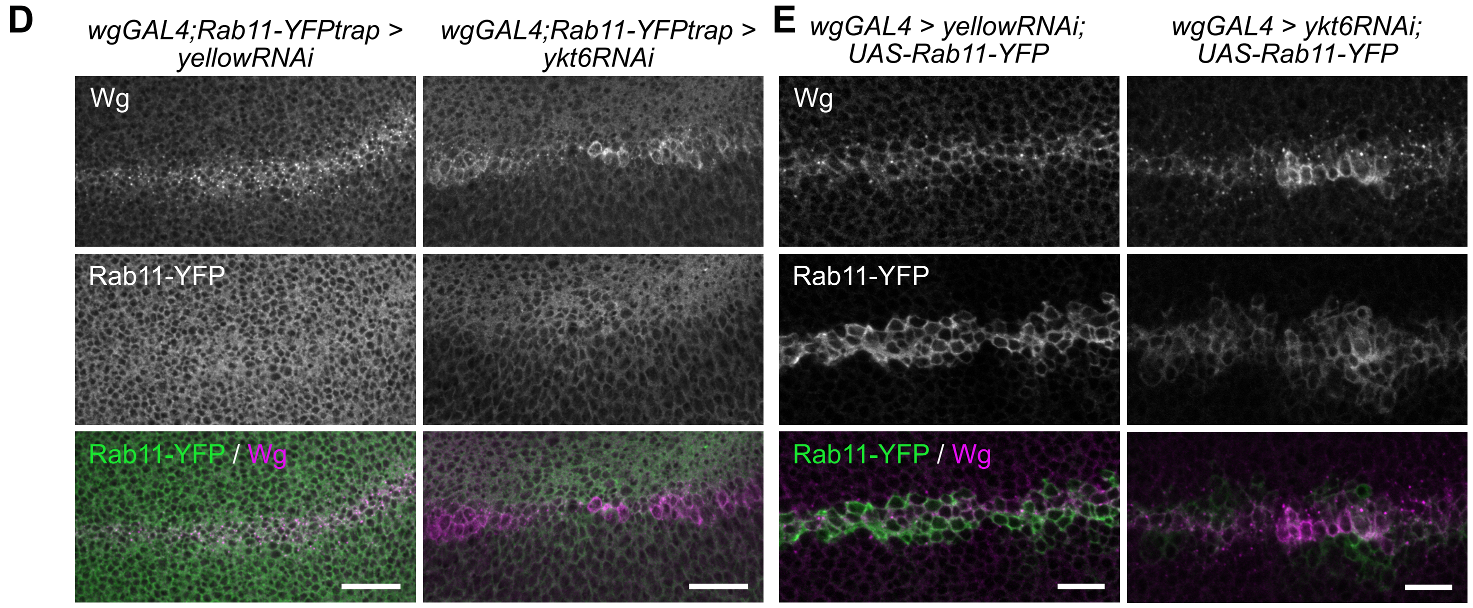

Fig. S6: Ykt6 SNARE domain is required in vivo. (**A**) Time-controlled depletion of Ykt6 by RNAi (engrailed-Gal4, UAS-GFP/ UAS-ykt6RNAi; tubGal80-TS/+, larvae reared for three days at days at 29°C) causes reduction of Senseless staining in the posterior WID. Images are representative of > six WIDs from three independent experiments. Scale bar: 20 µm (**B**) Depletion of ykt6 by RNAi along the dorso-ventral border using wingless-GAL4 results in adult wing notches. Wing notches can be rescued by UAS-driven co-overexpression of WT and SNARE mutant Ykt6-4A but not Ykt6-4E. All Ykt6 constructs are inserted on the third chromosome (86Fb). The images in the bottom panel show wings from the balancer flies from the same vial not co-expressing the UAS-Ykt6 constructs but only ykt6 RNAi as control. Scale bars 500 µm. These wings are representative of >10 wings from three independent experiments. (**C**) Rescue of lethality of *ykt6*^{A} and *ykt6*^{C} alleles. All Ykt6 constructs are inserted on the third chromosome (86Fb) and are expressed under the control of ubiquitous daughterless-GAL4. (**D**) yellow and ykt6 RNAi were expressed with wg-GAL4 in an endogenously tagged Rab11-YFP background. Maximum intensity projection of four sections (distance 0,5 µm) depicted for visualization. Scale bar represents 20 µm. (**E**) UAS-Rab11-YFP was expressed with wgGAL4 in combination with yellow (control, left panel) or ykt6 RNAi (right panel). Scale bars represent 20 µm.
