# Supplementary material for: Ykt6-dependent endosomal recycling is required for Wnt secretion in the Drosophila wing epithelium
Source: Development. 2020 Aug 14;147(15):dev185421. doi: 10.1242/dev.185421 (PMC7438013; doi:10.1242/dev.185421)
Supplement: Reviewer comments [file develop-147-185421_review_history.pdf]

Ykt6-dependent endosomal recycling is required for Wnt secretion in the *Drosophila* wing epithelium

Karen Linnemannstöns, Leonie Witte, Pradhipa Karuna M, Jeanette Clarissa Kittel, Adi Danieli, Denise Müller, Lena Nitsch, Mona Honemann-Capito, Ferdinand Grawe, Andreas Wodarz and Julia Christina Gross
DOI: 10.1242/dev.185421

Editor: Thomas Lecuit

Review timeline

Original submission:	9 October 2019
Editorial decision:	26 November 2019
First revision received:	11 April 2020
Editorial decision:	12 May 2020
Second revision received:	28 May 2020
Accepted:	8 June 2020

Original submission

First decision letter

MS ID#: DEVELOP/2019/185421

MS TITLE: Ykt6-endosomal recycling is required for Wnt secretion in the Drosophila wing epithelium

AUTHORS: Karen Linnemannstoens, Pradhipa Karuna M, Leonie Witte, Adi Danieli, Jeanette Clarissa Kittel, Denise Mueller, Lena Nitsch, Mona Honemann, Ferdinand Grawe, Andreas Wodarz, and Julia Christina Gross

I am sorry for the delay before being able to come back to you. However I have now received all the referees' reports on the above manuscript, and have reached a decision. The referees' comments are appended below, or you can access them online: please go to BenchPress and click on the 'Manuscripts with Decisions' queue in the Author Area.

As you will see, the referees express interest in your work, but have some criticisms and recommend a substantial revision of your manuscript before we can consider publication. If you are able to revise the manuscript along the lines suggested, which may involve further experiments, I will be happy receive a revised version of the manuscript. Your revised paper will be re-reviewed by one or more of the original referees, and acceptance of your manuscript will depend on your addressing satisfactorily the reviewers' major concerns. Please also note that Development will normally permit only one round of major revision.

Please attend to all of the reviewers' comments and ensure that you clearly highlight all changes made in the revised manuscript. Please avoid using 'Tracked changes' in Word files as these are lost in PDF conversion. I should be grateful if you would also provide a point-by-point response detailing how you have dealt with the points raised by the reviewers in the 'Response to Reviewers' box. If you do not agree with any of their criticisms or suggestions please explain clearly why this is so.

© 2020. Published by The Company of Biologists under the terms of the Creative Commons Attribution License (https://creativecommons.org/licenses/by/4.0/).

Reviewer 1

Advance summary and potential significance to field

In this manuscript the authors investigate the role of the SNARE Ykt6 in secretion of Wnt ligands, primarily studying Wg secretion in the Drosophila wing imaginal disc but with some comparison experiments in mammalian cell culture.
By comparison to Wg secretion phenotypes of other SNAREs, they show Ykt6 most likely acts around the same stage as Synaptobrevin (which acts at plasma membrane fusion in the later steps of secretion). Using BioID they show Ykt6 associates with endosomal proteins. One possibility would be that Ykt6 affects Wg secretion by reducing recycling of the Wnt cargo receptor Evi, however the authors present evidence arguing against this and instead pursue the hypothesis that Ykt6 acts in a post-Evi dependent step in Wg secretion. Through essentially indirect evidence (i.e. an accumulation of Wg outside Rab5-positive endosomes close to the plasma membrane upon Ykt6 knockdown, and a concomitant reduction in PI3P positive late endosomes), the authors infer that loss of Ykt6 may prevent fusion of Wg-containing endosome derived vesicles with the plasma membrane.
They further show that Ykt6 is normally localized to the cytosol but can reversibly associate with membranes via palmitoylation and argue it is recruited by acidified endosomes (although the experiments here need more explanation, at least for this reader), and that a functional SNARE domain is required for Ykt6 to adopt its soluble form and is also required for Wg secretion (although the data on this point are not very clear).
The final critical observations are that Wg is normally seen in Rab4-positive recycling endosomes and that Rab4 and Wg show increased colocalization at the plasma membrane upon Ykt6 RNAi, implicating Ykt6 in fusion of Rab4 endosomes at the plasma membrane. While this is plausible, I would be more convinced if these experiments used endogenously expressed Rab4 rather than overexpressed Rab4.
Overall, there are a lot of data in this manuscript, both genetics and biochemistry, and together they broadly support the authors' conclusions. The main weakness is that the key conclusions are largely inferred rather than demonstrated. To quote the abstract, I don't really think the authors can say definitely that 'most Ykt6 is inactive in the cytosol, but attaches to de-acidified compartments and recycles Wnts to the plasma membrane via Rab4-positive recycling endosomes'. There is certainly a large cytosolic population, but do we know it is inactive? Ykt6 may indeed by recruited to acidified endosomes, but are we really shown this (and can this be shown in vivo)? And can we be sure Wg recycles via Rab4-positive endosomes, when we only see them colocalizing upon Rab4 overexpression? These might seem to be unfair criticisms, given the difficulty of proving in vivo mechanisms, but all that is really needed is a bit more nuance in the way the authors present their conclusions i.e. tell us what you know for sure and what you don't know for sure.
With relatively straightforward revisions, I think this would make a strong contribution as an article in Development.

Comments for the author

Specific points:
• It might be better if the title started 'Ykt6-dependent endosomal recycling...' as the current title could be read to mean it is Ykt6 recycling (not Wnt recycling) that is important?
• Ref listed as 'Marois, 2005' should be 'Marois, Mahmoud and Eaton, 2006'?
• Fig.1G images do not clearly show a difference between the different RNAi phenotypes? Maybe the contrast needs increasing in the left-hand xy images so secreted Wg can be seen moving away from the DV boundary in 'wild-type' conditions (this isn't obvious in the review PDF). And/or the authors could do some quantification on the xz sections of a number of wings imaged on the same settings, to show the difference in intracellular accumulation between the A and P compartments.
• p.7 and Fig.2 – it is quite hard to work out what cells were used for the BioID. Given the lack of any indication, I initially assumed it was done in Drosophila wing discs, but the gene names in Fig.2 seem to be mammalian and the M&M says 'cells were seeded' but no cell line is specified. This information should ideally be in the main text, figure legend and M&M.
• Fig.4G – the authors make the important point that in ykt6-RNAi Wg labeling is seen outside of the Rab5[Q88L]-positive endosomes. The authors seem to suggest in the text that this Wg is in endosome-

© 2020. Published by The Company of Biologists under the terms of the Creative Commons Attribution License (https://creativecommons.org/licenses/by/4.0/).

derived secretory vesicles trapped below the plasma membrane because they are unable to fuse. While this may be true, I'm not sure why the extra-endosomal Wg labeling seems to be a strange stringy meshwork? Is this just cell outlines or are these some sort of tubular vesicles? Quite a lot of weight seems to be put on the interpretation of this one image. It would be helpful to have some higher resolution images (Airyscan? SIM?) and colabeling with membrane/vesicular markers to help to understand where this population of Wg is actually located. (See also comments regarding Fig.6.)
• Fig.5B,D,E and p.10 – based on the data in these panels, the authors apparently conclude that Ykt6 is recruited by acidified endosomes, but I had quite a lot of trouble following the logic. At face value, if Ykt6 is recruited by acidified endosomes, then inhibiting acidification with Bafilomycin should decrease Ykt6 recruitment, but in these experiments the opposite seems to happen? I'm also not sure what difference I'm supposed to see in the presence or absence of Palmostatin B. Also why does the blot in 5B shows cytoplasmic Ykt6 in all lanes, when only the last 4 have Ykt6 transfected, is there also detection of endogenous? And what are the stats for 5E, does ANOVA show any significance? It may be that these are quite standard biochemical assays and interpretations, but they probably need more justification for a developmental biology audience. Would it be possible to explain experiments in more detail in text and figure legends?
• Fig.5I-K – these rescue experiments would be a bit more convincing if the authors also showed the effects of introducing a 'benign' UAS construct in the same insertion site, as there are numerous examples of UAS-RNAi phenotypes being suppressed merely be the presence of additional UAS binding sites (that sequester GAL4). It is also puzzling that in the Ykt6-4E 'rescue' there is only increased Wg in a small spike some distance from the AP boundary (5J) – is this typical? Quantification across multiple independent wings might be a better way of presenting the data. This is also try of the wing notching phenotypes shown in 5H
– the example of a Ykt6-4E 'rescued' wing essentially has very little wing notching but instead seems to have distorted shape? Could this be due to a growth or cell morphology defect, rather than any effect on Wg secretion? (I note a similar experiment reappears in Fig.S6 which is more convincing, but somewhat confusing that the same point seems to be made twice?)
• Fig.6B and p.11-12 – similar to Fig.4G I think the authors are arguing that the relatively uniform distribution of Rab4-YFP and Wg at what seems to be cell edges is evidence of Wg trapped in secretory vesicles below the plasma membrane (and this is a bit more convincing here than in 4G). What is striking here is that the distribution doesn't look punctate (i.e. consistent with a vesicular localization). I realize that these cells are very small, but is there anything the authors could do to bolster the case that these are 'trapped' secretory vesicles? Admittedly EM would be difficult, but some form of super-resolution microscopy might help? Or at least colabeling with some other markers? I'm not sure it is helpful that the Rab4-YFP is overexpressed using the GAL4/UAS systems. Surely the same (or more clear?) results could be obtained with the endogenous Rab knock-ins (Dunst et al 2015)?
• Fig.S3 – legend suggests there are two panel Bs, but one of them seems to be missing?

<u>Reviewer 2</u>

Advance summary and potential significance to field

Ykt6-endosomal recycling is required for Wnt secretion in the Drosophila wing epithelium Karen Linnemannstöns et al., The authors re-evaluate the Wg phenotype of YKT6 loss of function obtained in their previous publication using both RNAi and validated with different mutant alleles.
Overall, the authors tested different possibilities to understand at which step YKT6 is necessary for Wg secretion. They propose that YKT6 is necessary for a secondary secretion of Wg, following its endocytosis.

Comments for the author

General notes

- Throughout the manuscript, it is often difficult to distinguish between signalling, secretion and trafficking. This is evident in the autocrine Wnt assay which analyses in the same time both Wnt secretion and signalling. Similarly, most of the initial hypothesis are based on the results of the

© 2020. Published by The Company of Biologists under the terms of the Creative Commons Attribution License (https://creativecommons.org/licenses/by/4.0/).

BioID approach, but the experimental design does not differentiate between secretory or endocytic pathways.

- The authors suggest that Ykt6 helps to recycle Wnt/Wg to the plasma membrane, yet no functional manipulation of recycling components is carried out. Additionally, no Wg/Wnt recycling assay was carried out.
The way in which the paper is structured makes it difficult to follow. There are also many spelling mistakes throughout the paper. In figure 4, there are two panels that have been repeated but labelled with two different genotypes.

- Low image quality, clearer representative images should be presented. Some of the experimental conditions used in the paper also appear to be quite harsh, and the authors do not provide cell viability controls.

- In several cases, the quantification methods are not appropriate to reveal granular defects/changes.
Also, normalisation of controls to 1 artificially reduces the variance of the data, increasing the risk of type 1 statistical error. This kind of normalisation has been carried out several times in the paper.

In conclusion, although this paper provides an insight into the conservation and function of the Ykt6 domains it does not build upon our understanding of Wg trafficking, or at least not sufficiently to convince this reviewer. The presented data do not generally support the conclusions presented by the authors.

Figure notes

Figure 1
Panel a: the anterior posterior boundary is not correctly labelled in this figure, possibly explaining why there is extracellular Wg staining in the posterior compartment. In this experiment and other in vivo experiments the authors used an en-gal4 which is visibly not exclusive to the posterior compartment of the wing imaginal disc.

Panel c: It is not clear what the highlighted square at the D/V is showing. Does it correspond to the inset shown below? Wingless is convincingly accumulated in the marked clones. Minor comment: the clone boundaries are not very precisely marked.
Panel d: The conclusion drawn in the text is not supported by the images presented. It would be important to show a quantification for the distribution of Wnt3A of these experiments. The cortical accumulation of Wnt3A is not visible in the presented image. Minor comment: include a zoom of the punctae to clarify the co-localisation of Wnt3A and YKT6?
Panel f: Only 1 RNAi line is used for each condition, it is difficult to compare the effects observed for the knock-down of each gene. Minor comments: the numbers of lines showing an effect on wingless in the text and figure do not match. The screen only looked at wing notching and wingless staining, did not look for cell lethality.

S1 Panel
a: The clones presented to the left of the image appear to be lacking nuclei.
Panel b: The sections presented for the cadherin staining are not at the right level in the disc. The sections appear to be lateral, but would need to be sub-apical.

Figure 2 Major concern: It is not made clear in the manuscript in which cell type the BioID was carried out.
This reviewer feels that the summaries of the BioID (Panels C, D and E) would be more appropriate in the sup.
Materials.
As the authors state, there was no interaction found with other snares. They suggest that this is due to a technical limitation. The authors have antibodies for the other snares, could have carried out CoIP to identify SNARE partners?

Figure 3

© 2020. Published by The Company of Biologists under the terms of the Creative Commons Attribution License (https://creativecommons.org/licenses/by/4.0/).

This is an important experiment considering the model presented. More thorough quantification would be needed to conclude that the loss of ykt6 function does not affect Evi distribution.

Panel a: This reviewer suggests that the authors present transverse sections of the Evi staining in a YKT6 KD condition. It appears the Evi is reduced in the posterior compartment under these conditions.
Panel b: The quantification methods here are not appropriate for detection of subtle changes in evi localisation/quantity. The average fluorescent intensity appears to have been measured, but the numbers of evi punctae appears to reduced. Loss of resolution due to quantification method.

Figure 4
Major comments: The authors show a general effect of ykt6 loss of function to the accumulation of Hrs, and show that removing one copy of hrs is sufficient to rescue the lethality associated with the ykt6 mutant, but do not link these two observations with the Wg phenotype.

Panel a: Major comment: The panels showing staining for Rab5 and Rab7 appear to be identical apart from a difference in the location of the A/P label. Contrary to what is stated in the text there does appear to be a difference in the anterior-posterior staining for either Rab5 or Rab7. Minor comment: The reference for the Rab5 antibody is missing from the materials and methods.

Sup figure 4A: The observation by the authors that removing one copy of hrs rescues the lethality of the ykt6 mutants is very interesting. Do the authors believe that it is the ykt6 dependent increase in hrs causing lethality in this context? To follow on from this point, does the over-expression of hrs lead to a Wg gain of function phenotype? Does the loss of one copy of hrs only rescue lethality, or does it also rescue the Wg trafficking defects observed in Ykt6 loss of function? Are the ykt6 wing phenotypes rescued by the loss of a copy of hrs?
It would be interesting to look at the effect of carrying out a Ykt6 RNAi with a wg-Gal4 in an hrs heterozygous background.
- What about wingless staining in the rescued condition? Is the lethality due to an impairment of signalling or a pleiotropic effect of the ykt6 loss of function?

Panel c: The maturation of MVBs does not appear to be affected in the knock-down of ykt6. To confirm this, it would be interesting to know whether the localisation of CHMP2B or ALIX are affected by the loss of ykt6.

Panel e: As PI3P is necessary for the recruitment of Hrs to early endosomes, it is surprising that there is an increase of hrs in a loss of ykt6. This result is not thoroughly addressed in the manuscript. It would be important to have a quantification of the numbers of FYVE labelled Wg punctae on transversal sections.

Panel g: minor comment: No UAS dosage control is provided. It would be interesting to see whether there is a change in the percentage or number of of Wg-containing Early endosomes in ykt6 knock-down. If there is a decrease in the percentage of EE containing Wg, this would suggest an endocytic defect. An increase in the percentage would suggest a trafficking defect.

Panel h: Minor comment: it is not clear what the image presented to the right represents.

Figure 5
Overall these data are convincing, but do not provide any insight as to the membrane domain in which ykt6 is active. It also also does not provide any insights as to how it affects Wg secretion.
Panel a: This reviewer finds it surprising that WT ykt6 is not observed in the membrane fraction.
Minor comment: the quantification suggests that this experiment was only carried out once.

Panel S5C: Could the authors provide a non-transfected control. How was co-localisation measured?
Panel S5D, E and F: Transfected cells appear to have reduced labelling for all the markers checked. The authors should check for cell viability in all the tested conditions.

Figure 6
General comments

© 2020. Published by The Company of Biologists under the terms of the Creative Commons Attribution License (https://creativecommons.org/licenses/by/4.0/).

The authors suggest that Ykt6 helps to recycle Wnt to the plasma membrane via Rab4, yet no functional manipulation of Rab4 is carried out.

Panel a: Could the authors comment on the heterogeneity of the wingless staining? The cells do not appear to be healthy in the Ykt6 knock-down.
Major comment: the authors state that “Expression of the slow recycling Rab11-YFP was not affected upon Ykt6 knockdown”. The provided image shows a strong change in Rab11-YFP distribution in the knockdown conditions. The staining appears more diffuse and the intensity is strongly reduced. This could either suggest
1) a qualitative change in the cells of the D/V boundary, 2) a change in the pattern of wg-gal4 expression, or
3) a change in the distribution of overexpressed Rab11-YFP. It would be interesting to carry out this experiment in a Rab11 knock-in background (available in the flybase collection).
Minor comment: in the manuscript the authors state that the expression of Rab11-YFP is not affected. This should rather be localisation.

Panel b: In these experiments Rab4 is being over-expressed. This reviewer would be interested to see the same treatments carried out in a Rab4 knock-in background. The phenotype that the Rab4 is re-localised in a Ykt6 knock-down does not establish a direct functional link between the Ykt6 and the fast recycling compartment. The pleiotropic nature of the loss of Ykt6 makes it difficult to interpret the results presented in this figure.
Panel6c: Minor comment: This reviewer is interested to know why the ykt6C mutant is not rescued by the UAS-Ykt6-4A.

minor comment: This experiment is lacking a UAS-control.

Reviewer 3

Advance summary and potential significance to field

This manuscript describes some important new insights into the secretion of Wingless (Wg) from signal-sending cells of the Drosophila wing disc. What is fascinating is that Wg is first sent to the apical plasma membrane, then endocytosed and trafficked to the basolateral domain to be secreted in the wing disc. The key findings are: (1) that Wg accumulates intracellularly in ykt6 mutant clones, seemingly at the plasma membrane, rather than being endocytosed and basally secreted; (2) that Wg accumulates together with Rab4 at the plasma membrane in the absence of ykt6, rather than being endocytosed into Rab4 positive endosomes and then subsequently secreted basally; (3) that mutation of the SNARE domain disrupts Ykt6 function. Thus, Ykt6 seems to have a function in trafficking of endocytosed Wg from the plasma membrane to Rab4 endosomes for subsequent secretion.

Comments for the author

These are all important findings and could be published in Development with consideration to the following points:

Perhaps it is worth citing the paper from Pascal Therond’s lab in Dev Cell (2015) on endocytosis of Hedgehog via Rab5 and Rab4 endosomes, particularly as they show that Rab4-RNAi or YFP-Rab4-dominant negative causes accumulation of Hh with Rab4 along basolateral plasma membranes. Did the authors try to express Rab4-RNAi or Rab4-DN and stain for Wg?

Page 13 “Wg and Rab4 partially colocalize in puncta, whereas both accumulate together intracellularly at the plasma membrane in Ykt6 RNAi, indicating that a Rab4 recycling step of Wg trafficking is mediated by Ykt6 fusion (Fig. 6B, C)”. Excellent, but doesn’t this once again predict that Rab4 RNAi or mutant cells should cause accumulation of Wg intracellularly?

Page 10: “Taken together, our in vivo genetic analyses demonstrate that Ykt6 is involved in fusion between endosomes and the plasma membrane during Wg trafficking.” Perhaps fusion is the wrong word here. I would instead consider stating that “Ykt6 appears to promote endocytic trafficking

© 2020. Published by The Company of Biologists under the terms of the Creative Commons Attribution License (https://creativecommons.org/licenses/by/4.0/).

from the plasma membrane towards Rab5 endosomes". Does this suggest a model similar to Hh, whereby endocytosed Wg enters Rab5 endosomes and is then sorted into Rab4 endosomes for subsequent secretion basolaterally (instead of returing to Rab11 endosomes for apical secretion)?

Fig 1D, the effect of siYkt6 on Wnt3A is not very clear… might be best to remove this data.

First revision

Author response to reviewers' comments

Thank you very much for your helpful and detailed comments, which we appreciate very much. Please find our point-by-point reply to the reviewer comments on DEVELOP/2019/185421 now termed "**Ykt6-dependent endosomal recycling is required for Wnt secretion in the Drosophila wing epithelium**". Our replies are highlighted in blue underneath the particular reviewer comments. We performed several additional experiments confirming our previous argumentation that Ykt6 acts in a post-Evi- dependent step in Wg secretion.

These includes:

- A Wg recycling assay shows that endocytosis of Wg is not affected by Ykt6 KD.
- More detailed analysis of Wg in Rab4-endosomes, including endogenous rab4/Wg colocalization and effects of functional inhibition of Rab4, which increased apical localization of Wg and rab5.
- Hrs/Ykt6 genetic interaction:
    - o There is no Wg gain of function phenotype in Hrs-OE nor in Hrs-KD.
    - o Hrs protein stability in Kc167 is not significantly altered.
    - o Rab5/Wg colocalization is decreased upon Ykt6 KD.

Moreover, we added several missing control experiments and changed the quantification and normalization of WID as suggested by the reviewers:
1. UAS- and yellow KD controls are confirming the previous findings/quantification.
2. Airy scan imaging confirms accumulation of Wg at the cortext near plasma membrane, but below DE-cadherin junctions.

According to these results, we have changed the figures and text, detailed responses are given below and are highlighted in yellow in the manuscript. We hope to have achieved significant improvement of our manuscript to make it suitable for publication in Development.

Point-by-point reply to the reviewer comments on DEVELOP/2019/185421:

Reviewer 1 Advance Summary and Potential Significance to Field:
In this manuscript the authors investigate the role of the SNARE Ykt6 in secretion of Wnt ligands, primarily studying Wg secretion in the Drosophila wing imaginal disc but with some comparison experiments in mammalian cell culture.
By comparison to Wg secretion phenotypes of other SNAREs, they show Ykt6 most likely acts around the same stage as Synaptobrevin (which acts at plasma membrane fusion in the later steps of secretion). Using BioID they show Ykt6 associates with endosomal proteins. One possibility would be that Ykt6 affects Wg secretion by reducing recycling of the Wnt cargo receptor Evi, however the authors present evidence arguing against this and instead pursue the hypothesis that Ykt6 acts in a post-Evi dependent step in Wg secretion. Through essentially indirect evidence (i.e. an accumulation of Wg outside Rab5- positive endosomes close to the plasma membrane upon Ykt6 knockdown, and a concomitant reduction in PI3P positive late endosomes), the authors infer that loss of Ykt6 may prevent fusion of Wg-containing endosome derived vesicles with the plasma membrane.
They further show that Ykt6 is normally localized to the cytosol but can reversibly associate with membranes via palmitoylation and argue it is recruited by acidified endosomes (although the experiments here need more explanation, at least for this reader), and that a functional

© 2020. Published by The Company of Biologists under the terms of the Creative Commons Attribution License (https://creativecommons.org/licenses/by/4.0/).

SNARE domain is required for Ykt6 to adopt its soluble form and is also required for Wg secretion (although the data on this point are not very clear).
The final critical observations are that Wg is normally seen in Rab4-positive recycling endosomes and that Rab4 and Wg show increased colocalization at the plasma membrane upon Ykt6 RNAi, implicating Ykt6 in fusion of Rab4 endosomes at the plasma membrane. While this is plausible, I would be more convinced if these experiments used endogenously expressed Rab4 rather than overexpressed Rab4.

1.1) We thank the reviewer for his/her constructive feedback and address several of his concerns. We added additional experiments to show that Ykt6 is involved in a secondary secretion step from endosomes seen in novel Fig 6 and S6. These more direct approaches include a Wg recycling assay (Fig. S3, see also response 2.3), endogenous Rab4 labeling (Fig. 6, see also response 1.15) and we also added more detailed explanation for the cell culture /biochemical assay in novel Fig 5. More details are provided below.

Overall, there are a lot of data in this manuscript, both genetics and biochemistry, and together they broadly support the authors' conclusions. The main weakness is that the key conclusions are largely inferred rather than demonstrated. To quote the abstract, I don't really think the authors can say definitely that 'most Ykt6 is inactive in the cytosol, but attaches to de- acidified compartments and recycles Wnts to the plasma membrane via Rab4-positive recycling endosomes'. There is certainly a large cytosolic population, but do we know it is inactive?

1.2) We agree with the reviewer that our data does not prove that cytosolic Ykt6 is inactive, but this conclusion rather stems from published biochemical and structural Ykt6 studies (Fukasawa et al., 2004; Tochio, 2001). A mutation in the longin domain (position F42A) prevents its intramolecular interaction with the SNARE domain and C-terminal farnesylation to fold into a soluble, inactive conformation (Fukasawa et al., 2004; Tochio, 2001). We changed the text to state more carefully that "…most Ykt6 is present in the cytosol, but can be recruited to de-acidified compartments…"(line 37-38).

Ykt6 may indeed by recruited to acidified endosomes, but are we really shown this (and can this be shown in vivo)?

1.3) We apologize for the confusion. We propose that Ykt6 is recruited to de-acidified compartments, thus to endosomal compartments trafficking towards the plasma membrane rather than more acidic degradative endosomes. We clarified this aspect in the text. Our endeavor to show Ykt6 recruitment *in vivo* was unsuccessful, as a generated Ykt6 antibody does not work in immunostaining of WID. In addition, we repeated the cell culture membrane recruitment assay with different reagents blocking acidification (NH4Cl, Chloroquine) and see in both cases, that - similar to bafilomycin A1 - blocking acidification of endosomes while blocking depalmitoylation at the same time leads to membrane recruitment of Ykt6 in Hek293T cells (novel Figure 5F and G).

And can we be sure Wg recycles via Rab4-positive endosomes, when we only see them colocalizing upon Rab4 overexpression?

1.4) See response no. 1.15.

These might seem to be unfair criticisms, given the difficulty of proving in vivo mechanisms, but all that is really needed is a bit more nuance in the way the authors present their conclusions i.e. tell us what you know for sure and what you don't know for sure. With relatively straightforward revisions, I think this would make a strong contribution as an article in Development.

Reviewer 1 Comments for the Author: Specific points:

- *It might be better if the title started 'Ykt6-dependent endosomal recycling...' as the current title could be read to mean it is Ykt6 recycling (not Wnt recycling) that is important?*

1.5) We changed the title accordingly (line 1).

© 2020. Published by The Company of Biologists under the terms of the Creative Commons Attribution License (https://creativecommons.org/licenses/by/4.0/).

• *Ref listed as 'Marois, 2005' should be 'Marois, Mahmoud and Eaton, 2006'?*

1.6) We changed the reference accordingly (line 70).

• *Fig.1G images do not clearly show a difference between the different RNAi phenotypes? Maybe the contrast needs increasing in the left-hand xy images so secreted Wg can be seen moving away from the DV boundary in 'wild-type' conditions (this isn't obvious in the review PDF). And/or the authors could do some quantification on the xz sections of a number of wings imaged on the same settings, to show the difference in intracellular accumulation between the A and P compartments.*

1.7) We apologize for the bad quality of the PDF. We have exchanged the images for maximum intensity projections for better visualization, now seen in novel Figure 1F. We changed the figure legend accordingly.

• *p.7 and Fig.2 – it is quite hard to work out what cells were used for the BioID. Given the lack of any indication, I initially assumed it was done in Drosophila wing discs, but the gene names in Fig.2 seem to be mammalian and the M&M says 'cells were seeded' but no cell line is specified. This information should ideally be in the main text, figure legend and M&M.*

1.8) We apologize for this omission, and changed the text (line 162), figure legend and M&M accordingly (line 583).

• *Fig.4G – the authors make the important point that in ykt6-RNAi Wg labeling is seen outside of the Rab5[Q88L]-positive endosomes. The authors seem to suggest in the text that this Wg is in endosome-derived secretory vesicles trapped below the plasma membrane because they are unable to fuse. While this may be true, I'm not sure why the extra-endosomal Wg labeling seems to be a strange stringy meshwork? Is this just cell outlines or are these some sort of tubular vesicles? Quite a lot of weight seems to be put on the interpretation of this one image. It would be helpful to have some higher resolution images (Airyscan? SIM?) and colabeling with membrane/vesicular markers to help to understand where this population of Wg is actually located. (See also comments regarding Fig.6.)*

1.9) We co-labeled ykt6 knock-down WID with DE-cadherin and Wg and performed Airy scan imaging in novel SupFig 4E. We observe that the membranous Wg signal at the cortex is below the DE-Cad domain (no colocalization). See also response 1.14.

• *Fig.5B,D,E and p.10 – based on the data in these panels, the authors apparently conclude that Ykt6 is recruited by acidified endosomes, but I had quite a lot of trouble following the logic. At face value, if Ykt6 is recruited by acidified endosomes, then inhibiting acidification with Bafilomycin should decrease Ykt6 recruitment, but in these experiments the opposite seems to happen? I'm also not sure what difference I'm supposed to see in the presence or absence of Palmostatin B. Also why does the blot in 5B shows cytoplasmic Ykt6 in all lanes, when only the last 4 have Ykt6 transfected, is there also detection of endogenous? And what are the stats for 5E, does ANOVA show any significance? It may be that these are quite standard biochemical assays and interpretations, but they probably need more justification for a developmental biology audience. Would it be possible to explain experiments in more detail in text and figure legends?*

1.10) We apologize for the confusion. Ykt6 is recruited to de-acidified compartments. Thus, Bafilomycin should increase Ykt6 to channel cargo into recycling pathways. PalmostatinB prevents de-palmitoylation and this should arrest Ykt6 at these membranes, further increasing the Ykt6 levels in the membrane fraction, as seen in novel Fig.5F and G. We provide more details about these sets of experiments in the text and figure legends. In addition, we now show several different inhibitors of acidification to shift Ykt6 into the membranes fraction in combination with Palmostatin B, see response 1.3 and novel Figure 5 F-I .

• *Fig.5I-K – these rescue experiments would be a bit more convincing if the authors also showed the effects of introducing a 'benign' UAS construct in the same insertion site, as there are numerous examples of UAS-RNAi phenotypes being suppressed merely be the presence of*

© 2020. Published by The Company of Biologists under the terms of the Creative Commons Attribution License (https://creativecommons.org/licenses/by/4.0/).

additional UAS binding sites (that sequester GAL4).

1.11) We have repeated the cross with a benign UAS construct: enGAL4; tubGAL80TS X ykt6KK; Tsp96F
This construct used the same vector backbone and is inserted in the same attP landing site ZH-86Fb (BL 24749). The result is in line with the previous conclusion and is shown in novel Fig. 5 J, L, N.

It is also puzzling that in the Ykt6-4E 'rescue' there is only increased Wg in a small spike some distance from the AP boundary (5J) – is this typical? Quantification across multiple independent wings might be a better way of presenting the data.

1.12) We thank the reviewer for this comment and have exchanged the fluorescence profiles for quantifications of the average fluorescence intensity in the novel Fig. 5 J-O. This way the anterior side of the enGAL4 WID serves as an internal control and the fold change of the posterior (RNAi) side across several discs can be measured. See also response no. 2.6. regarding statistical details.

This is also try of the wing notching phenotypes shown in 5H – the example of a Ykt6-4E 'rescued' wing essentially has very little wing notching but instead seems to have distorted shape? Could this be due to a growth or cell morphology defect, rather than any effect on Wg secretion? (I note a similar experiment reappears in Fig.S6 which is more convincing, but somewhat confusing that the same point seems to be made twice?)

1.13) It makes a difference if enGAL4 or wgGAL4 is used: ykt6 knockdown with wgGAL4 makes clear notches, which can be rescued by WT Ykt6 and Ykt6-4A, but not by Ykt6-4E (Fig. S6). However, the wgGAL4 driver does not allow to compare anterior vs posterior WID. Ykt6 knockdown with enGAL4 is lethal, which is why we switched to a milder time-controlled KD using GAL80TS. Using this experimental setting, we did observe growth defects, when the larvae were reared too long at 29°C. This shows that Ykt6 KD leads to more defects than only blocking Wg secretion. Therefore, we only analyzed WIDs without overall cell defects for Wg secretion defects. 4E for sure does not rescue these additional functions, leading to the distorted wing shape. The reviewer is completely right that the effect on Wg secretion might be a consequence of this.

• *Fig.6B and p.11-12 – similar to Fig.4G I think the authors are arguing that the relatively uniform distribution of Rab4-YFP and Wg at what seems to be cell edges is evidence of Wg trapped in secretory vesicles below the plasma membrane (and this is a bit more convincing here than in 4G). What is striking here is that the distribution doesn't look punctate (i.e. consistent with a vesicular localization). I realize that these cells are very small, but is there anything the authors could do to bolster the case that these are 'trapped' secretory vesicles? Admittedly EM would be difficult, but some form of super-resolution microscopy might help? Or at least colabeling with some other markers?*

1.14) See also response 1.9. We co-labeled ykt6 knockdown WID with DE-cadherin and Wg and performed Airy scan imaging. We observe that the membranous Wg signal at the cortex is below the DE-Cadherin domain, so there is no apical colocalization, trapped Wg rather localizes laterally, below the membrane, as seen in novel Figure S4E.

I'm not sure it is helpful that the Rab4-YFP is overexpressed using the GAL4/UAS systems. Surely the same (or more clear?) results could be obtained with the endogenous Rab knock-ins (Dunst et al 2015)?

1.15) The reviewer is absolutely right that endogenous YFP traps are superior to overexpressed UAS constructs. However, the genomic location of Rab4 is on the second chromosome, as well as the wgGAL4/ enGAL4 driver and ykt6 RNAi lines. We instead crossed the Rab4-YFP trap together with MS1096GAL4 on the first chromosome (expressed in the WID pouch), but unfortunately ykt6 knockdown is lethal using this GAL4 driver. But in the control situation (MS1096GAL4; Rab4-YFPtrap > yellow RNAi) endogenous Rab4-YFP looks similar to overexpressed UAS-Rab4-YFP, which is why we assume that UAS- Rab4-YFP still gives us valid information on the

© 2020. Published by The Company of Biologists under the terms of the Creative Commons Attribution License (https://creativecommons.org/licenses/by/4.0/).

function of Rab4. We included this information in the text (line 345-350) and novel Fig. 6A.

- *Fig.S3 – legend suggests there are two panel Bs, but one of them seems to be missing?*

1.16) We apologize for the confusion and corrected the legend accordingly.

Reviewer 2 Advance Summary and Potential Significance to Field:
Ykt6-endosomal recycling is required for Wnt secretion in the Drosophila wing epithelium Karen Linnemannstöns et al., The authors re-evaluate the Wg phenotype of YKT6 loss of function obtained in their previous publication using both RNAi and validated with different mutant alleles. Overall, the authors tested different possibilities to understand at which step YKT6 is necessary for Wg secretion. They propose that YKT6 is necessary for a secondary secretion of Wg, following its endocytosis.

Reviewer 2 Comments for the Author: General notes
-Throughout the manuscript, it is often difficult to distinguish between signalling, secretion and trafficking. This is evident in the autocrine Wnt assay which analyses in the same time both Wnt secretion and signalling. Similarly, most of the initial hypothesis are based on the results of the BioID approach, but the experimental design does not differentiate between secretory or endocytic pathways.

2.1) We took care to more precisely define whether an experiment affects Wg signaling, secretion or trafficking changed the text and figure legend accordingly. Our conclusions are based on two lines of evidence: (1) The Ykt6 effect on Wg staining in the Wnt secreting cells with different endosomal components (Ap2, SNX3, Hrs). (2) The effect of Ykt6 and AP2 knockdown on Evi (Fig. 3A-C), as an important carrier of the secretory pathway. Because Ykt6 had no strong effect on Evi, we concluded that Ykt6 acts on the secretion of Wg from endosomal compartments, after separation from Evi, as we clearly see a difference in extracellular Wg levels in Ykt6 knockdown (Fig. 3D-F).

-The authors suggest that Ykt6 helps to recycle Wnt/Wg to the plasma membrane, yet no functional manipulation of recycling components is carried out.

2.2) We added several additional experiments, investigating Wg/Wnt recycling by impairing Rab4 and Rab11 function and summarized the results in a table at the end of the rebuttal letter. While Rab11 knockdown was lethal, Rab4 KD in one of three tested RNAi lines had a significant effect on Wg transport (novel Fig 6D-F), this RNAi line was also causing accumulation of Hh in the paper D'Angelo Dev Cell 2015. Neither expression of UAS-Rab4-DN or UAS-Rab4-CA in Wg expressing cells affected localization of Wg nor resulted in adult wing notches, but these rab4 stainings did not correspond to endogenous Rab4 patterns. See also response 3.3.

Additionally, no Wg/Wnt recycling assay was carried out.

2.3). With a Wg antibody-uptake assay we did not observe impaired apical endocytosis of Wg upon Ykt6 KD (now novel Fig S3). This is in line with experiments in Fig. 3 that AP2 KD has a different effect on extracellular Wg and Evi/WLS than Ykt6 KD. Therefore, we see the endocytosis assay as a confirmation that Ykt6 is neither involved in primary secretion nor endocytosis of Wg.

The way in which the paper is structured makes it difficult to follow. There are also many spelling mistakes throughout the paper. In figure 4, there are two panels that have been repeated but labelled with two different genotypes.

2.4) We apologize for these mistakes and changed the images to the correct images for each phenotype, see also response no. 2.21.

-Low image quality, clearer representative images should be presented. Some of the experimental conditions used in the paper also appear to be quite harsh, and the authors do not provide cell viability controls.

© 2020. Published by The Company of Biologists under the terms of the Creative Commons Attribution License (https://creativecommons.org/licenses/by/4.0/).

2.5) Image acquisition was carefully conducted regarding bit depth, saturation, offset and dynamic range. For ykt6 RNAi experiments we used a GAL80 time controlled mild knockdown system to circumvent any manipulation that might be too harsh. We have exchanged several images and refer to response no. 1.7, 2.7, 2.14 and 2.28. Regarding experimental conditions and cell viability controls we refer to response no. 2.13 and 2.33.

-In several cases, the quantification methods are not appropriate to reveal granular defects/changes. Also, normalisation of controls to 1 artificially reduces the variance of the data, increasing the risk of type 1 statistical error. This kind of normalisation has been carried out several times in the paper.

2.6) For enGAL4RNAi WID experiments we have used two types of quantification: normalized intensity of a representative example as in Figure 3B and averaged intensity for five or more WID, as seen in a recent Wg paper (Chaudhary and Boutros Development 2019). Because variation in staining intensity and imaging is high and the anterior side of enGAL4 WID serves as an internal control, we consider the fold change of the RNAi side over several discs to be a good indication for a robust biological effect. To circumvent the statistical disadvantage of losing variance in the control by normalizing to 1, we have now performed normalization to the mean of all measurement per WID. Where applicable, we quantified particles (novel Fig. 3C).

In conclusion, although this paper provides an insight into the conservation and function of the Ykt6 domains, it does not build upon our understanding of Wg trafficking, or at least not sufficiently to convince this reviewer. The presented data do not generally support the conclusions presented by the authors.

Figure notes
Figure 1
Panel a: the anterior posterior boundary is not correctly labelled in this figure, possibly explaining why there is extracellular Wg staining in the posterior compartment. In this experiment and other in vivo experiments the authors used an en-gal4 which is visibly not exclusive to the posterior compartment of the wing imaginal disc.

2.7) The image depicted a maximum intensity projection and the AP border is not perfectly straight in the xz plane. Not to confuse the reader and also referring to point 2.5, we have inserted a clearer representative image in the novel Fig. 1A. We now correctly labelled the AP boundary with a manually drawn line and have also included more details in the novel figure legend as highlighted.

Panel c: It is not clear what the highlighted square at the D/V is showing. Does it correspond to the inset shown below?

2.8) We apologize for this omission and changed the figure legend accordingly. The changes are highlighted in the text.

Wingless is convincingly accumulated in the marked clones. Minor comment: the clone boundaries are not very precisely marked.

2.9) We marked the clone boundaries more precisely.

Panel d: The conclusion drawn in the text is not supported by the images presented. It would be important to show a quantification for the distribution of Wnt3A of these experiments. The cortical accumulation of Wnt3A is not visible in the presented image. Minor comment: include a zoom of the punctae to clarify the co-localisation of Wnt3A and YKT6?

2.10) As reviewer 3 suggested, we took out these human cell images and changed the figure legend accordingly.

Panel f: Only 1 RNAi line is used for each condition, it is difficult to compare the effects observed for the knock-down of each gene.

© 2020. Published by The Company of Biologists under the terms of the Creative Commons Attribution License (https://creativecommons.org/licenses/by/4.0/).

2.11) The reviewer is right that we used only one RNAi line for the primary screen looking for Wing notches. Interesting candidates, which had notches, were further analyzed. The secondary screen was conducted with several RNAi lines per candidates. We have added this additional information in Supplementary Table 2.

Minor comments: the numbers of lines showing an effect on wingless in the text and figure do not match.

2.12) We cordially apologize for this confusion and changed the numbers in the figure and text accordingly (line 138).

The screen only looked at wing notching and wingless staining, did not look for cell lethality.

2.13) We agree with the reviewer, that knockdown of SNAREs has pleiotropic effects, possibly leading to cell lethality. It is correct that for those candidates that had notches in the primary screen and then were lethal with enGAL4 in the secondary screen, the notches might have been caused by cell death. For this reason, we did not analyze these candidates any further. Wingless staining/expression was only analyzed when the whole disc was overall intact without any apoptotic cells, as judged by Hoechst co-staining. Upon reviewer's request, we can provide overview images of all SNARE knockdowns and of every experimental condition tested.

S1
Panel a: The clones presented to the left of the image appear to be lacking nuclei.

2.14) We have exchanged the image for a new image in which all nuclei within the GFP-negative clones are clearly Hoechst- positive.

Panel b: The sections presented for the cadherin staining are not at the right level in the disc. The sections appear to be lateral, but would need to be sub-apical.

2.15) We agree with the reviewer that the quality of the DE-Cadherin antibody staining was not appropriate and have exchanged the image for a clearer image. The section presented for the DE-Cadherin staining now is subapical and the section presented for Wingless is lateral as indicated in the novel figure legend S1B.

Figure 2
Major concern: It is not made clear in the manuscript in which cell type the BioID was carried out. This reviewer feels that the summaries of the BioID (Panels C, D and E) would be more appropriate in the sup. Materials.

2.16) We apologize for this omission, and changed the text, figure legend and M&M accordingly (line 162). The Panels D and E of former Fig 2 are now included in novel Fig. S3A, B. and described in the Supplementary figure legend.

As the authors state, there was no interaction found with other snares. They suggest that this is due to a technical limitation. The authors have antibodies for the other snares, could have carried out CoIP to identify SNARE partners?

2.17) Previous studies of Ykt6 focusing on understanding its function via its interacting SNARE partners (McNew et al., 1997; Zhang and Hong, 2001, Fukasawa et al., 2004, Gordon et al., 2017) found it to be involved in different SNARE complexes, that is why we chose to take a novel approach, such as BioID labeling.

Figure 3
This is an important experiment considering the model presented. More thorough quantification would be needed to conclude that the loss of ykt6 function does not affect Evi distribution. Panel a: This reviewer suggests that the authors present transverse sections of the Evi staining in a YKT6 KD condition. It appears the Evi is reduced in the posterior compartment under these

© 2020. Published by The Company of Biologists under the terms of the Creative Commons Attribution License (https://creativecommons.org/licenses/by/4.0/).

conditions.

2.18) We have included the corresponding transverse section. In our opinion, this does not reveal reduced Evi in the posterior compartment. See also response no. 2.19.

Panel b: The quantification methods here are not appropriate for detection of subtle changes in evi localisation/ quantity. The average fluorescent intensity appears to have been measured, but the numbers of evi punctae appears to reduced. Loss of resolution due to quantification method.

2.19) We have quantified the number of Evi punctae inYkt6 RNAi versus control. However, in comparison to AP2alpha RNAi we only see a slight and not significant decrease in Evi punctae upon Ykt6 knockdown. In contrast, in 3D-F we depicted the extracellular Wg, which is reduce upon ykt6 KD, as shown in Fig. 1A.

Figure 4
Major comments: The authors show a general effect of ykt6 loss of function to the accumulation of Hrs, and show that removing one copy of hrs is sufficient to rescue the lethality associated with the ykt6 mutant, but do not link these two observations with the Wg phenotype.

2.20) We refer to response 2.23 and 2.24.

Panel a: Major comment: The panels showing staining for Rab5 and Rab7 appear to be identical apart from a difference in the location of the A/P label. Contrary to what is stated in the text there does appear to be a difference in the anterior-posterior staining for either Rab5 or Rab7.

2.21) We cordially apologize for this mistake and changed the images accordingly. We are aware that upon initial visual inspection there might be a difference in the anterior vs posterior staining and that is why we quantified several images.

Minor comment: The reference for the Rab5 antibody is missing from the materials and methods.

2.22) We have included the Rab5 antibody reference in the materials and methods section (line 480-481).

Sup figure 4A: *The observation by the authors that removing one copy of hrs rescues the lethality of the ykt6 mutants is very interesting. Do the authors believe that it is the ykt6 dependent increase in hrs causing lethality in this context?*

2.23) Hrs KD leads to an expansion of Wg distribution, published in Seto and Bellen, JCB 2006. See next point.

To follow on from this point, does the over-expression of hrs lead to a Wg gain of function phenotype?

2.24) Similarly to Hrs KD, when we overexpressed UAS-Hrs with wgGAL4, this leads to intracellular Wg accumulation and is lethal at pupal stage, a Wg loss of function phenotype (Seto & Bellen, 2006). Moreover, enGal4 driven UAS-Hrs is lethal. We summarized these results in a table at the end of this letter. This effect suggest, that Hrs positive endosomes are the turning point of Wg trafficking. Hrs can sort cargo into MVBs for degradation or promote cargo recycling. In Kc167 cells there is no significant effect of Ykt6 KD on HRS protein levels (see additional Figure 1).

We have removed unpublished data provided for the referees in confidence.

Additional Figure 1: Western Blot from Kc cells: quantification of protein level of Hrs and Ykt6 are not depend on each other. Knockdown of Ykt6 levels does not change Hrs protein levels compared to control RNAi, while Hrs knockdown does not influence Ykt6 stability compared to control, experiment was performed in triplicates, standard deviation and students t-test

© 2020. Published by The Company of Biologists under the terms of the Creative Commons Attribution License (https://creativecommons.org/licenses/by/4.0/).

significance levels*= 0.02, ***=0.0001.

Does the loss of one copy of hrs only rescue lethality, or does it also rescue the Wg trafficking defects observed in Ykt6 loss of function? Are the ykt6 wing phenotypes rescued by the loss of a copy of hrs? It would be interesting to look at the effect of carrying out a Ykt6 RNAi with a wg-Gal4 in an hrs heterozygous background. -What about wingless staining in the rescued condition? Is the lethality due to an impairment of signalling, or a pleiotropic effect of the ykt6 loss of function?

2.25) We have tried to combine Ykt6 and Hrs RNAi to compare it with Yellow and Hrs RNAi (enGFP;tubGAL80TS/CyO X ykt6KK; Hrs TRiP 33900 vs. enGFP;tubGAL80TS/CyO X yellowKK; Hrs TRiP 33900). This would have allowed us, to compare the interesting rescue phenotype, but unfortunately, we were not successful in obtaining the fly lines expressing both RNAi constructs in time. If the reviewer feels our initial observation is too preliminary, we can exclude this data (Fig. S4A) from the current manuscript.

Panel c: The maturation of MVBs does not appear to be affected in the knock-down of ykt6. To confirm this, it would be interesting to know whether the localisation of CHMP2B or ALIX are affected by the loss of ykt6.

2.26) *Drosophila* Alix antibody (Haglund, Stenmark) did not work in our hands and human antibodies do not recognize the
Drosophila protein.

Panel e: As PI3P is necessary for the recruitment of Hrs to early endosomes, it is surprising that there is an increase of hrs in a loss of ykt6. This result is not thoroughly addressed in the manuscript. It would be important to have a quantification of the numbers of FYVE labelled Wg punctae on transversal sections.

2.27) We thank the reviewer for this suggestion and have inserted transversal sections with quantification of the number of FYVE labelled Wg punctae in the novel figure 4 F and H. We have changed the figure legend and text accordingly.
Panel g: minor comment: No UAS dosage control is provided.

2.28) We have exchanged the wgGAL4 X Rab5Q88L images for images of the genotype wgGAL4; Rab5Q88L X yellow and did the quantification accordingly (novel Fig. 4I and J). We changed the figure legend accordingly.

It would be interesting to see whether there is a change in the percentage or number of of Wg-containing Early endosomes in ykt6 knock-down. If there is a decrease in the percentage of EE containing Wg, this would suggest an endocytic defect. An increase in the percentage would suggest a trafficking defect.

2.29) We have done Rab5-Wg colocalization analysis in Fig. S4I. We indeed observe a decrease in Rab5-Wg colocalization upon ykt6 RNAi. Together with the finding that in Ykt6 KD Wg endocytosis is unchanged, but colocalization with FYVE-GFP is decreased and colocalization with Rab4 increased, this argues for a Wg accumulation before secondary secretion from recycling endosomes. We changed the text and figure legend accordingly.

Panel h: Minor comment: it is not clear what the image presented to the right represents.

2.30) This panel is a control cross to show the phenotype of Wg accumulation in wg-Gal4 driven Ykt6 RNAi. We moved this image to Fig. S4D and changed the legends accordingly.

Figure 5
Overall these data are convincing, but do not provide any insight as to the membrane domain in which ykt6 is active. It also also does not provide any insights as to how it affects Wg secretion. Panel a: This reviewer finds it surprising that WT ykt6 is not observed in the membrane fraction.

© 2020. Published by The Company of Biologists under the terms of the Creative Commons Attribution License (https://creativecommons.org/licenses/by/4.0/).

2.31) Our endeavor to identify the organelle membrane *in vivo*, to which Ykt6 gets recruited was unsuccessful, as a generated Ykt6 antibody does not work in immunostaining of WID. In human Hek293T cells, we found endosomal proteins in our BioID approach. Membrane recruitment was demonstrated biochemically in Hek293 cells, with Ykt6 overexpression, in control lanes low levels of endogenous Ykt6 are seen. Our cell fractionation experiments confirm the published findings of Ykt6 cytosol to membrane cycling and indicates that the majority of Ykt6 is the cytoplasmic form. The steady state level of Ykt6 in the membrane fractions is below the detection limit of WB, in line with the observation that most WT-Ykt6 is not palmitoylated and therefore cytoplasmic as seen in Figure 5C.

Minor comment: the quantification suggests that this experiment was only carried out once.

2.32) We apologize for this omission. This experiment was performed six times and we added this in the figure legend of novel Fig. 5A.

Panel S5C: Could the authors provide a non-transfected control. How was co-localisation measured?
Panel S5D, E and F: Transfected cells appear to have reduced labelling for all the markers checked. The authors should check for cell viability in all the tested conditions.

2.33) This experiment was performed to supplement the membrane fractionation experiments in the main figure 5. Localization of overexpressed Ykt6 was inspected by eye, rather than quantified by colocalization correlation to give some localization information in non-polarized human cells. There were no obvious differences in cell numbers in control and Ykt6 immunofluorescence slides.

Figure 6
General comments
The authors suggest that Ykt6 helps to recycle Wnt to the plasma membrane via Rab4, yet no functional manipulation of Rab4 is carried out.

2.34) As suggested by the reviewer, we performed Rab4 knockdown, now included in novel Fig 6 D-F. See also response 2.2 and 3.1.

Panel a: Could the authors comment on the heterogeneity of the wingless staining? The cells do not appear to be healthy in the Ykt6 knock-down.

2.35) Ykt6 knockdown by wgGAL4 leads to Wg secretion defect. In contrast to Evi knockdown where no Wg is secreted at all, the effect is not equally pronounced in all cells along the Wg stripe. Some cells do secrete and some have high intracellular Wg levels. The wing imaginal discs are healthy as they develop into adult wings with small notches. As mentioned in response no. 2.13 Wingless staining/expression was only analyzed when the whole disc was overall intact without any apoptotic cells, as judged by Hoechst co-staining. Upon reviewer's request, we can provide the Hoechst panel as well as images of adult wings.

Major comment: the authors state that "Expression of the slow recycling Rab11-YFP was not affected upon Ykt6 knockdown". The provided image shows a strong change in Rab11-YFP distribution in the knockdown conditions. The staining appears more diffuse and the intensity is strongly reduced. This could either suggest 1) a qualitative change in the cells of the D/V boundary, 2) a change in the pattern of wg-gal4 expression, or 3) a change in the distribution of overexpressed Rab11-YFP. It would be interesting to carry out this experiment in a Rab11 knock-in background (available in the flybase collection).

2.36) The reviewer is absolutely right that endogenous YFP traps are superior to overexpressed UAS constructs. We have therefore crossed the Rab11-YFP trap together with wgGAL4. In this scenario, the control situation (wgGAL4; Rab11-YFPtrap > yellow RNAi) endogenous Rab11-YFP localization is similar to the ykt6 knock down (wgGAL4; Rab11-YFPtrap > ykt6 RNAi). We have moved both Rab11-YFP trap and UAS-Rab11-YFP experiments to novel Fig. S6 D,E and changed text (line 342) and figure legends accordingly.

© 2020. Published by The Company of Biologists under the terms of the Creative Commons Attribution License (https://creativecommons.org/licenses/by/4.0/).

Minor comment: in the manuscript the authors state that the expression of Rab11-YFP is not affected. This should rather be localisation.

2.37) The reviewer is absolutely right and we changed the text accordingly (line 342).

Panel b: In these experiments Rab4 is being over-expressed. This reviewer would be interested to see the same treatments carried out in a Rab4 knock-in background. The phenotype that the Rab4 is re-localised in a Ykt6 knock-down does not establish a direct functional link between the Ykt6 and the fast recycling compartment. The pleiotropic nature of the loss of Ykt6 makes it difficult to interpret the results presented in this figure.

2.38) As also suggested by Reviewer 1, we have included endogenous Rab4-YFP trap in our analysis and refer to response 1.15.

Panel6c: Minor comment: This reviewer is interested to know why the ykt6C mutant is not rescued by the UAS-Ykt6-4A.

2.39) The two ykt6 alleles differ in that they one carries a point mutation in the Longin domain and the other in the start codon. We sequenced the stocks and realized that the annotation at Flybase/Bloomington is not correct:

- ***ykt6^A*** (BL57143), annotated as M1I in fact carries **Q62R**, the mutation in the Longin domain.
- ***ykt6^C*** (BL57142), annotated as Q62R in fact carries **M1I**, the mutation in the start codon.

The detailed information is provided in Supplementary Information and we have corrected Figure 1G accordingly.

We speculate that the *ykt6^C* allele represents a full loss-of-function due to the mutated start codon. This cannot be rescued by UAS-Ykt6-4A. *ykt6^A* on the other hand appears to be a hypomorphic allele. The mutation in the Longin domain alone is lethal, but can be rescued with UAS-Ykt6-4A.

minor comment: This experiment is lacking a UAS-control.

2.40) We have repeated the cross with a benign UAS construct: ykt6A; daGAL4 X ykt6KK; Tsp42Ef. This construct used the same vector backbone and is inserted in the same attP landing site ZH-86Fb (BL 24749). The result is shown in novel Fig. S6C.

Reviewer 3 Advance Summary and Potential Significance to Field:
This manuscript describes some important new insights into the secretion of Wingless (Wg) from signal-sending cells of the Drosophila wing disc. What is fascinating is that Wg is first sent to the apical plasma membrane, then endocytosed and trafficked to the basolateral domain to be secreted in the wing disc. The key findings are: (1) that Wg accumulates intracellularly in ykt6 mutant clones, seemingly at the plasma membrane, rather than being endocytosed and basally secreted;
(2) that Wg accumulates together with Rab4 at the plasma membrane in the absence of ykt6, rather than being endocytosed into Rab4 positive endosomes and then subsequently secreted basally; (3) that mutation of the SNARE domain disrupts Ykt6 function. Thus, Ykt6 seems to have a function in trafficking of endocytosed Wg from the plasma membrane to Rab4 endosomes for subsequent secretion.
Reviewer 3 Comments for the Author:
These are all important findings and could be published in Development with consideration to the following points:
Perhaps it is worth citing the paper from Pascal Therond's lab in Dev Cell (2015) on endocytosis of Hedgehog via Rab5 and Rab4 endosomes, particularly as they show that Rab4-RNAi or YFP-Rab4-dominant negative causes accumulation of Hh with Rab4 along basolateral plasma membranes.

3.1) We thank the reviewer for his positive comments. Indeed, we cited the very

© 2020. Published by The Company of Biologists under the terms of the Creative Commons Attribution License (https://creativecommons.org/licenses/by/4.0/).

important paper of Pascal Therond's lab (Dev Cell 2015) in the discussion (line 395).

Did the authors try to express Rab4-RNAi or Rab4-DN and stain for Wg?

3.2) Please see also response 2.2. Rab4 KD in one of three tested RNAi lines had a significant effect on Wg transport (novel Fig 6D-F), this RNAi line was also causing accumulation of Hh in the paper of D'Angelo (Dev Cell 2015). Neither expression of UAS-Rab4-DN or UAS-Rab4-CA in Wg expressing cells affected localization of Wg nor resulted in adult wing notches, but these rab4 stainings did not correspond to endogenous Rab4 patterns. In addition, it is possible, that redundancy of Rab4- and Rab11- dependent recycling makes it difficult to observe stronger Wg phenotype with Rab4 KD only. Alternatively, Wg secretion via Rab4 endosomes could be a compensatory mechanism of Wg release under Ykt6 KD conditions.

Page 13 "Wg and Rab4 partially colocalize in puncta, whereas both accumulate together intracellularly at the plasma membrane in Ykt6 RNAi, indicating that a Rab4 recycling step of Wg trafficking is mediated by Ykt6 fusion (Fig. 6B, C)". Excellent, but doesn't this once again predict that Rab4 RNAi or mutant cells should cause accumulation of Wg intracellularly?

3.3) See above, response 3.2. We have performed the suggested experiments in novel Figure 6.

Page 10: "Taken together, our in vivo genetic analyses demonstrate that Ykt6 is involved in fusion between endosomes and the plasma membrane during Wg trafficking." Perhaps fusion is the wrong word here. I would instead consider stating that "Ykt6 appears to promote endocytic trafficking from the plasma membrane towards Rab5 endosomes". Does this suggest a model similar to Hh, whereby endocytosed Wg enters Rab5 endosomes and is then sorted into Rab4 endosomes for subsequent secretion basolaterally (instead of returing to Rab11 endosomes for apical secretion)?

3.4) Similar to Hh secondary secretion, it seems that Wg is endocytosed apical and is resecreted via Rab5-Rab4 endosomes. D'Angelo and colleagues showed that Hh is redistributed basolaterally in the absence of Rab4, while we see an intracellular apical accumulation of Wg in the absence of Ykt6. But due to the different effect on Wg and Evi in AP2 and Ykt6 RNAi and the lack of difference in Wg endocytosis between control and Ykt6 KD in the Wg recycling assay (novel Fig S3) , we concluded that Ykt6 affects Wg only after endocytosed Wg and Evi have separated in endosomes (Coombs, Virshup, JCS 2010).

Fig 1D, the effect of siYkt6 on Wnt3A is not very clear… might be best to remove this data.

3.5) We agree with the reviewer and reviewer 2 on this point and have removed Fig. 1D, see also comment no. 2.10.

List of experiments for revision that are not included in the figures IF wing imaginal discs:

	Fly cross performed	Staining	Replicates	Results
**Rab4 /Rab11 functional**				
**Rab4 RNAi**	enGFP; UAS-Dcr X Rab4 TRiP 33757	Wg	3	no phenotype
	enGFP; UAS-Dcr X Rab4 KK106651	Wg, Rab5	3	apical accumulation, intermediate
	enGFP; UAS-Dcr X Rab4 GD24672	Wg, Rab5	3	no phenotype
**Rab11 RNAi**	enGFP; UAS-Dcr X Rab11 TRiP 27730	NA	NA	lethal at 25°C, RT, 18°C
	enGFP X Rab11 TRiP 27730	NA	NA	lethal at 25°C, RT, 18°C

© 2020. Published by The Company of Biologists under the terms of the Creative Commons Attribution License (https://creativecommons.org/licenses/by/4.0/).

	enGFP; UAS-Dcr X Rab11 dsRNA BL42790	Wg	2	no phenotype
**Rab4 DN/CA**	wgGAL4/CyOtwiGFP X UAS-YFP-Rab4Q67L III (BL 9770)	Wg, Rab4	3	cytoplasmic, Rab4 not punctate, Wg
	wgGAL4/CyOtwiGFP X UAS-YFP-Rab4S22N II (BL9768)	Wg, Rab4	3	cytoplasmic, Rab4 not punctate, Wg
**Connection between Ykt6**				
**Hrs RNAi**	UAS-Dcr; enGAL4,UASGFP X Hrs TRiP 33900	Wg	3	Wg distribution affected, as in Seto & Bellen, 2006
	UAS-Dcr; enGAL4,UASGFP X Hrs TRiP 28026	Wg	3	Wg distribution affected, as in Seto & Bellen, 2006
	UAS-Dcr; enGAL4,UASGFP X Hrs TRiP 34086	Wg	3	Wg distribution affected, as in Seto & Bellen, 2006
**Hrs OE**	wgGAL4/CyOtwiGFP X UAS-Hrs (BL 42692)	Wg	3	Intracellular Wg
	enGFP/CyO X UAS-Hrs (BL 42692)	NA	NA	lethal at 25°C, RT, 18°C

Second decision letter

MS ID#: DEVELOP/2019/185421

MS TITLE: Ykt6-dependent endosomal recycling is required for Wnt secretion in the Drosophila wing epithelium

AUTHORS: Karen Linnemannstoens, Leonie Witte, Pradhipa Karuna M, Adi Danieli, Jeanette Clarissa Kittel, Denise Mueller, Lena Nitsch, Mona Honemann, Ferdinand Grawe, Andreas Wodarz, and Julia Christina Gross

I have now received all the referees reports on the above manuscript, and have reached a decision. The referees' comments are appended below, or you can access them online: please go to BenchPress and click on the 'Manuscripts with Decisions' queue in the Author Area.

The overall evaluation is positive and we would like to publish a revised manuscript in Development, provided that the referees' comments can be satisfactorily addressed. I wish to stress the the manuscript indeed needs very thorough editing as detailed by reviewer 1. Please attend to all of the reviewers' comments in your revised manuscript and detail them in your point-by-point response.

We are aware that you may currently be unable to access the lab to undertake experimental revisions. If it would be helpful, we encourage you to contact us to discuss your revision in greater detail. Please send us a point-by-point response indicating where you are able to address concerns raised (either experimentally or by changes to the text) and where you will not be able to do so within the normal timeframe of a revision. We will then provide further guidance. Please also note that we are happy to extend revision timeframes as necessary.
However in this case since all revisions simply involve a thorough edition of the text, I do not expect any delay due to the current epidemics.

© 2020. Published by The Company of Biologists under the terms of the Creative Commons Attribution License (https://creativecommons.org/licenses/by/4.0/).

Reviewer 1

Advance summary and potential significance to field

See previous review.

Comments for the author

There's a lot of work here and I think the results (just about) justify the conclusions, so I think it should be published. However, the manuscript is still a hard read (and not simply because the subject area is a complex one, although this doesn't help). I've listed below things I noticed that seemed to be either wrong confusing or apparent over/mis-interpretations (many of which I no doubt missed the first time around).
These can of course all be fixed, but ideally the authors should not just make these specific changes, but give the whole manuscript a careful revision (and maybe ask some colleagues to carefully read it too), to try to make it as clear and easy to read as possible. The value of the work to the community will be rather diminished if readers struggle to follow the arguments or get disillusioned due to seeing repeated errors or confusing statements.
Specific points:

- Is the summary statement actually a summary of the results in the manuscript? It seems more like a general statement about the importance of Wg resecretion, which most would argue has been previously shown by other groups?
- Line 34 'Why the cell undergoes these trafficking steps' – it is of course Wg that undergoes the trafficking, not the cell
- Line 70, should be 'Marois et al., 2006', not 'Marois, 2006'
- Line 76, 'Frz' is a undefined abbreviation, presumably for Frizzled. However the ligand is Wg (a Drosophila protein) and the Drosophila abbreviation for Frizzled is 'Fz'
- Line 140, 'Syb' not defined
- Lines 151-153, 'knockdown of Ykt6 and VAMP1 (human Syb homologue) reduced Wnt activity whereas Sec22B and VAMP7 did not (Supplementary Fig. 1E)' – legend says 'Not significant, one-way ANOVA' for these data, which seems to imply no pair-wise comparisons showed any significant difference? Could the stated 'results' just be noise? Or do pair-wise comparisons show significant differences? (I'm not sure that's possible if the omnibus test shows no significance?)
- Line 175 '(46)' is a mis-formatted citation?
- Lines 181-182, 'reduced autocrine Wnt signaling activity (Supplementary Fig. 2D)' – again legend says
'Not significant, one-way ANOVA' , again does this imply there are no statistically significant changes in this experiment?
- Lines 201-202, 'we found that Ykt6 knockdown had no effect on Evi (Fig. 3A-C), thus ruling out a function of Ykt6 in Evi recycling' – I'm not sure why the authors conclude 'no effect' rather than a 'weak effect'? Quantitation in 3B shows a significant effect of Ykt6 RNAi, and 3C shows a strong trend in the same direction (and without a power calculation, 'not significant' can't be taken to mean 'no effect'). Given this is RNAi (and KD may not be of equal efficiency), without further investigation surely the only safe conclusion is that both AP2 and Ykt6 KD affect Evi? (Also not sure why switch from 'Evi' to 'Wls' on y-axes between 3B and 3C.)
- Line 194, 'Ykt6 is not required for Evi recycling' – see previous point, at best the conclusion would have to be that the Ykt6 KD (which appears weaker than the loss-of-function clone phenotype) has some effect on Evi levels (and hence recycling?).
- Lines 235, 1035, 1060, should be 'puncta' not 'punctae' (also y-axes in 3C, 4G)
- Lines 245-246, 'Wg was seen outside Rab5Q88L endosomes close to the apical membrane (Fig. 4I, right panel and J)' – I don't understand how we know that the Wg that isn't in endosomes in these images is close to the apical membrane. Should there be an xz section here too? The strange tubular appearance of the Wg-labeled structures remains mysterious.
- Lines 247-248, 'Higher resolution using Airyscan imaging revealed that Wg accumulates below junctions as marked with DE-Cadherin (Supplementary Fig. 4E)' – the text (and the response to reviewers) implies Fig.S4E is the same genotype as Fig.4I (or at least is Ykt6 RNAi), but this isn't reflected in the legend to Fig.S4E. I'm also not sure the authors have interpreted the images in S4E correctly. Without an xz section it is hard to know, but in the apical section, Wg seems to be in the apical membranes *above* the adherens junctions, then as you go below the adherens junctions you still see Wg outlining the cell edges, consistent with a population of Wg either in lateral membranes

© 2020. Published by The Company of Biologists under the terms of the Creative Commons Attribution License (https://creativecommons.org/licenses/by/4.0/).

or in vesicles closely associated with lateral membranes. This interpretation is also consistent with the xz section in Fig.S4H where there is strong lateral Wg labeling.
• Line 254, 'Supplementary Fig. 4I' – '4H' presumably?
• Lines 254-255, 'our in vivo genetic analyses demonstrate that Ykt6 is involved in Wg trafficking between endosomes and the plasma membrane' – I really am nervous about the authors saying they have
'demonstrated' this at this point. I think it's quite hard to know where the Wg population seen upon Ykt6 RNAi is localized, and where it has come from or is going. The only reason this conclusion makes sense, is that the Wg is seen to colocalize with Rab4, but we haven't been shown this result yet.
• Lines 346-347, 'Both an endogenously tagged Rab4-YFP and overexpressed UAS-Rab4-YFP partially colocalize in puncta under control conditions (Fig. 6 A,B)' – I think the authors mean that both look punctate, rather than that they colocalize with each other (which is not shown and anyway wouldn't be possible to show unless one had a different tag). I'm not sure how we know these puncta are the same structures?
• Line 352, 'Rab5' not 'rab5'
• Lines 352-353, 'In agreement with this recycling route, in RNAi of Rab4 Wg and rab5 accumulated towards the apical membrane (Fig.6 D-F)' – presumably they should colocalize, as Wg should now be trapped in a Rab5 positive early recycling endosome? However, image is far too small to see much (and we aren't shown an overlay).
• Lines 356, 432, 'Rab4' not 'rab4'
• Lines 879-880, need to add other authors
• Lines 903-904, unlikely this was published in volume 0? (My eye happened to fall on this error, but it would be good if the authors checked the reference list for other errors.)
• Line 1018 (also line 156), 'Fig. 2: Ykt6 acts in Wg endocytosis after apical presentation' – seems like a strange heading/figure title. The manuscript argues that Ykt6 acts in Wg re-secretion after endocytosis which I'm not sure is the same as acting in Wg endocytosis. Or have I missed something? Is there a second function of Ykt6 in endocytosis?
• Line 1031, 'Fig. 3: Ykt6 is not required for Evi recycling.' – as noted earlier, based on the evidence presented, there *is* an effect of Ykt6 RNAi on Evi levels, and so it seems hard to rule out an effect on Evi recycling
• Line 1075, again for Fig.5E we're told 'One-way Anova, significance level: n.s.' – does this mean no significant differences in pair-wise comparisons in this experiment?
• Lines 1080, 1083, here one-way ANOVA shows significance, but the authors must have done a post-hoc pairwise test (Tukey's?) to determine specific differences? Can they clarify and explain in Materials and Methods?

Reviewer 3

Advance summary and potential significance to field

The manuscript provides new insights into trafficking of Wg via Ykt6 and Rab4 endosomes prior to secretion.

Comments for the author

I am satisfied with the revised manuscript and now recommend publication.

Second revision

Author response to reviewers' comments

Thank you very much for considering the revised version of our manuscript DEVELOP/2019/185421 termed **"Ykt6-dependent endosomal recycling is required for Wnt secretion in the Drosophila wing epithelium"**. Please find our point-by-point reply to reviewer 1 comments. Our detailed

© 2020. Published by The Company of Biologists under the terms of the Creative Commons Attribution License (https://creativecommons.org/licenses/by/4.0/).

responses are written in blue underneath the particular reviewer comments and are highlighted in yellow in the manuscript. Additionally, we edited the manuscript to remove misleading points as suggested by reviewer 1. We highlighted these changes in green in the manuscript.

By this we hope to have achieved significant improvement of our manuscript to make it suitable for publication in Development.

Best regards,

Point-by-point reply to the reviewer comments on DEVELOP/2019/185421:

Reviewer 1 specific points:

1. Is the summary statement actually a summary of the results in the manuscript? It seems more like a general statement about the importance of Wg resecretion, which most would argue has been previously shown by other groups?

We changed the summary statement to “SNARE-dependent secondary secretion via Rab4 recycling endosomes play an important role in coordinating long-range extracellular levels of the hydrophobic morphogen Wnt/Wingless.”

2. Line 34 ‘Why the cell undergoes these trafficking steps’ – it is of course Wg that undergoes the trafficking, not the cell

We corrected the text from “the cell” to “Wnt molecules”.

3. Line 70, should be ‘Marois et al., 2006’, not ‘Marois, 2006’

We corrected the reference accordingly.

4. Line 76, ‘Frz’ is a undefined abbreviation, presumably for Frizzled. However the ligand is Wg (a Drosophila protein) and the Drosophila abbreviation for Frizzled is ‘Fz’

We corrected the text accordingly.

5. Line 140, ‘Syb’ not defined

We included the full definition “Synaptobrevin (Syb)” in the text.

6. Lines 151-153, ‘knockdown of Ykt6 and VAMP1 (human Syb homologue) reduced Wnt activity whereas Sec22B and VAMP7 did not (Supplementary Fig. 1E)’ – legend says ‘Not significant, one-way ANOVA’ for these data, which seems to imply no pair-wise comparisons showed any significant difference? Could the stated ‘results’ just be noise? Or do pair-wise comparisons show significant differences? (I’m not sure that’s possible if the omnibus test shows no significance?)

As these knockdowns were done in one experiment, we used one-way ANOVA with multiple comparison as the correct way for statistical testing. With high standard deviations of some siRNA (SNAP25 and STX8), the significance is lost, even though in some cases the signaling decrease to >50% of the control in the case of Ykt6 or VAMP1. Pairwise comparison by t-test of these siRNA shows significant differences. If requested we can add -although not significantly- in line 147“…knockdown of Ykt6 and VAMP1 (human Syb homologue) reduced Wnt activity (although not significantly), whereas Sec22B and VAMP7 did not.”

7. Line 175 ‘(46)’ is a mis-formatted citation?

Yes. We apologize and corrected the text accordingly to “Roux et al., 2018”.

8. Lines 181-182, ‘reduced autocrine Wnt signaling activity (Supplementary Fig. 2D)’ – again legend says ‘Not significant, one-way ANOVA’ , again does this imply there are no statistically significant changes in this experiment?

© 2020. Published by The Company of Biologists under the terms of the Creative Commons Attribution License (https://creativecommons.org/licenses/by/4.0/).

See point 6. If requested we can add - although not significantly - in line 176: "Furthermore, knockdown of Dynamin2, Chmp2B and Alix in Hek293T Wnt reporter cells reduced (although not significantly) autocrine Wnt signaling activity (Supplementary Fig. 2D)."

9. Lines 201-202, 'we found that Ykt6 knockdown had no effect on Evi (Fig. 3A-C), thus ruling out a function of Ykt6 in Evi recycling' – I'm not sure why the authors conclude 'no effect' rather than a 'weak effect'? Quantitation in 3B shows a significant effect of Ykt6 RNAi, and 3C shows a strong trend in the same direction (and without a power calculation, 'not significant' can't be taken to mean 'no effect'). Given this is RNAi (and KD may not be of equal efficiency), without further investigation surely the only safe conclusion is that both AP2 and Ykt6 KD affect Evi?

We agree with this critical point and changed the text to "we found that Ykt6 knockdown only had a weak effect on Evi (Fig. 3A-C), thus making a function of Ykt6 in Evi recycling unlikely".

(Also not sure why switch from 'Evi' to 'Wls' on y-axes between 3B and 3C.)

We labelled the y-axes uniformly with "Evi".

10. Line 194, 'Ykt6 is not required for Evi recycling' – see previous point, at best the conclusion would have to be that the Ykt6 KD (which appears weaker than the loss-of-function clone phenotype) has some effect on Evi levels (and hence recycling?).

We changed the title of the paragraph and the corresponding figure legend to "Ykt6 knockdown is not sufficient to block Evi recycling"

11. Lines 235, 1035, 1060, should be 'puncta' not 'punctae' (also y-axes in 3C, 4G)

We corrected the text accordingly.

12. Lines 245-246, 'Wg was seen outside Rab5Q88L endosomes close to the apical membrane (Fig. 4I, right panel and J)' – I don't understand how we know that the Wg that isn't in endosomes in these images is close to the apical membrane. Should there be an xz section here too? The strange tubular appearance of the Wg-labeled structures remains mysterious.

The reviewer is correct and we apologize for the imprecise wording and the resulting misunderstanding. Wg accumulates at the membrane along the entire z axis. We therefore removed the word "apical" from the text.

13. Lines 247-248, 'Higher resolution using Airyscan imaging revealed that Wg accumulates below junctions as marked with DE-Cadherin (Supplementary Fig. 4E)' – the text (and the response to reviewers) implies Fig.S4E is the same genotype as Fig.4I (or at least is Ykt6 RNAi), but this isn't reflected in the legend to Fig.S4E.

It is the same genotype as Fig. S4D (Ykt6 RNAi). We apologize for this omission and corrected the figure legend accordingly.

I'm also not sure the authors have interpreted the images in S4E correctly. Without an xz section it is hard to know, but in the apical section, Wg seems to be in the apical membranes *above* the adherens junctions, then as you go below the adherens junctions you still see Wg outlining the cell edges, consistent with a population of Wg either in lateral membranes or in vesicles closely associated with lateral membranes. This interpretation is also consistent with the xz section in Fig.S4H where there is strong lateral Wg labeling.

The reviewer is correct and we apologize for the imprecise wording and the resulting misunderstanding. We changed the text to: "Airyscan imaging revealed that Wg accumulates mainly laterally below junctions as marked with DE- Cadherin."

14. Line 254, 'Supplementary Fig. 4I' – '4H' presumably?

© 2020. Published by The Company of Biologists under the terms of the Creative Commons Attribution License (https://creativecommons.org/licenses/by/4.0/).

We refer to the colocalization analysis of Wg and endogenous Rab5 as depicted in Fig. S4I. To make this more conclusive we moved the reference and the text now reads: "Colocalization of Wg- and endogenous Rab5 was significantly decreased in ykt6 KD (Supplementary Fig. 4I) further confirming Rab5Q88L results."

15. Lines 254-255, 'our in vivo genetic analyses demonstrate that Ykt6 is involved in Wg trafficking between endosomes and the plasma membrane' – I really am nervous about the authors saying they have 'demonstrated' this at this point. I think it's quite hard to know where the Wg population seen upon Ykt6 RNAi is localized, and where it has come from or is going. The only reason this conclusion makes sense, is that the Wg is seen to colocalize with Rab4, but we haven't been shown this result yet.

We changed the text to "our *in vivo* genetic analyses demonstrate that Ykt6 is involved in endosomal Wg trafficking required for Wnt release" and bring up the membrane association aspect in the next paragraph. Here, we made some additional changes highlighted in green, see below for details.

16. Lines 346-347, 'Both an endogenously tagged Rab4-YFP and overexpressed UAS-Rab4-YFP partially colocalize in puncta under control conditions (Fig. 6 A,B)' – I think the authors mean that both look punctate, rather than that they colocalize with each other (which is not shown and anyway wouldn't be possible to show unless one had a different tag). I'm not sure how we know these puncta are the same structures?

The sentence is supposed to read "Both an endogenously tagged Rab4-YFP and overexpressed UAS-Rab4-YFP partially colocalize with Wg in puncta under control conditions." We corrected the missing word and apologize for this omission and the resulting misunderstanding.

17. Line 352, 'Rab5' not 'rab5'

We corrected the text accordingly.

18. Lines 352-353, 'In agreement with this recycling route, in RNAi of Rab4 Wg and rab5 accumulated towards the apical membrane (Fig.6 D-F)' – presumably they should colocalize, as Wg should now be trapped in a Rab5 positive early recycling endosome? However, image is far too small to see much (and we aren't shown an overlay).

Fig. 4E and F are the quantifications of Wg and Rab5 fluorescence intensity of the images as presented in Fig. 4D. Based on this quantification there is a (significant) shift towards the apical region of both proteins and this is what we state in the text. The reviewer is absolutely right that the two proteins presumably colocalize there, but this would be an over-interpretation of the data at this point. Therefore, we edited the text to: "Wg accumulated towards the apical membrane (Fig. 6 D, E), similar to Rab5 (Fig. 6 D, F).

19. Lines 356, 432, 'Rab4' not 'rab4'

We corrected the text accordingly.

20. Lines 879-880, need to add other authors

We apologize for the incomplete reference and have added the missing authors.

21. Lines 903-904, unlikely this was published in volume 0? (My eye happened to fall on this error, but it would be good if the authors checked the reference list for other errors.)

We corrected the reference accordingly and also checked the entire reference list for errors.

22. Line 1018 (also line 156), 'Fig. 2: Ykt6 acts in Wg endocytosis after apical presentation' – seems like a strange heading/figure title. The manuscript argues that Ykt6 acts in Wg re-secretion after endocytosis, which I'm not sure is the same as acting in Wg endocytosis. Or have I missed something? Is there a second function of Ykt6 in endocytosis?

© 2020. Published by The Company of Biologists under the terms of the Creative Commons Attribution License (https://creativecommons.org/licenses/by/4.0/).

The reviewer is right that this subtitle might be misleading the reader. We changed title of the paragraph and the corresponding figure legend to "Ykt6 acts on endosomal compartments after apical presentation".

23. Line 1031, 'Fig. 3: Ykt6 is not required for Evi recycling.' – as noted earlier, based on the evidence presented, there *is* an effect of Ykt6 RNAi on Evi levels, and so it seems hard to rule out an effect on Evi recycling

See above. We changed the title of the paragraph and the corresponding figure legend to "Ykt6 knockdown is not sufficient to block Evi recycling".

24. Line 1075, again for Fig.5E we're told 'One-way Anova, significance level: n.s.' – does this mean no significant differences in pair-wise comparisons in this experiment?

Yes, see 6. for detailed comment.

25. Lines 1080, 1083, here one-way ANOVA shows significance, but the authors must have done a post-hoc pairwise test (Tukey's?) to determine specific differences? Can they clarify and explain in Materials and Methods?

We performed one-way ANOVA with a post-hoc Dunnett's multiple comparison test as the standard procedure proposed by Prism 8 to compare a control mean with the other means, and have included this information in the Material and Methods.

Third decision letter

MS ID#: DEVELOP/2019/185421

MS TITLE: Ykt6-dependent endosomal recycling is required for Wnt secretion in the Drosophila wing epithelium

AUTHORS: Karen Linnemannstoens, Leonie Witte, Pradhipa Karuna M, Adi Danieli, Jeanette Clarissa Kittel, Denise Mueller, Lena Nitsch, Mona Honemann, Ferdinand Grawe, Andreas Wodarz, and Julia Christina Gross
ARTICLE TYPE: Research Article

I read your reponse to the few remaining comments from one of the reviewers and in light of your revisions I am happy to tell you that your manuscript has been accepted for publication in Development, pending our standard ethics checks.

© 2020. Published by The Company of Biologists under the terms of the Creative Commons Attribution License (https://creativecommons.org/licenses/by/4.0/).
